# Temporal Decoding of MAP Kinase and CREB Phosphorylation by Selective Immediate Early Gene Expression

**DOI:** 10.1371/journal.pone.0057037

**Published:** 2013-03-04

**Authors:** Takeshi H. Saito, Shinsuke Uda, Takaho Tsuchiya, Yu-ichi Ozaki, Shinya Kuroda

**Affiliations:** 1 Department of Biophysics and Biochemistry, Graduate School of Science, University of Tokyo, Bunkyo-ku, Tokyo, Japan; 2 CREST, Japan Science and Technology Corporation, Bunkyo-ku, Tokyo, Japan; The University of Tokyo, Japan

## Abstract

A wide range of growth factors encode information into specific temporal patterns of MAP kinase (MAPK) and CREB phosphorylation, which are further decoded by expression of immediate early gene products (IEGs) to exert biological functions. However, the IEG decoding system remain unknown. We built a data-driven based on time courses of MAPK and CREB phosphorylation and IEG expression in response to various growth factors to identify how signal is processed. We found that IEG expression uses common decoding systems regardless of growth factors and expression of each IEG differs in upstream dependency, switch-like response, and linear temporal filters. Pulsatile ERK phosphorylation was selectively decoded by expression of EGR1 rather than c-FOS. Conjunctive NGF and PACAP stimulation was selectively decoded by synergistic JUNB expression through switch-like response to c-FOS. Thus, specific temporal patterns and combinations of MAPKs and CREB phosphorylation can be decoded by selective IEG expression via distinct temporal filters and switch-like responses. The data-driven modeling is versatile for analysis of signal processing and does not require detailed prior knowledge of pathways.

## Introduction

**Figure 1:**
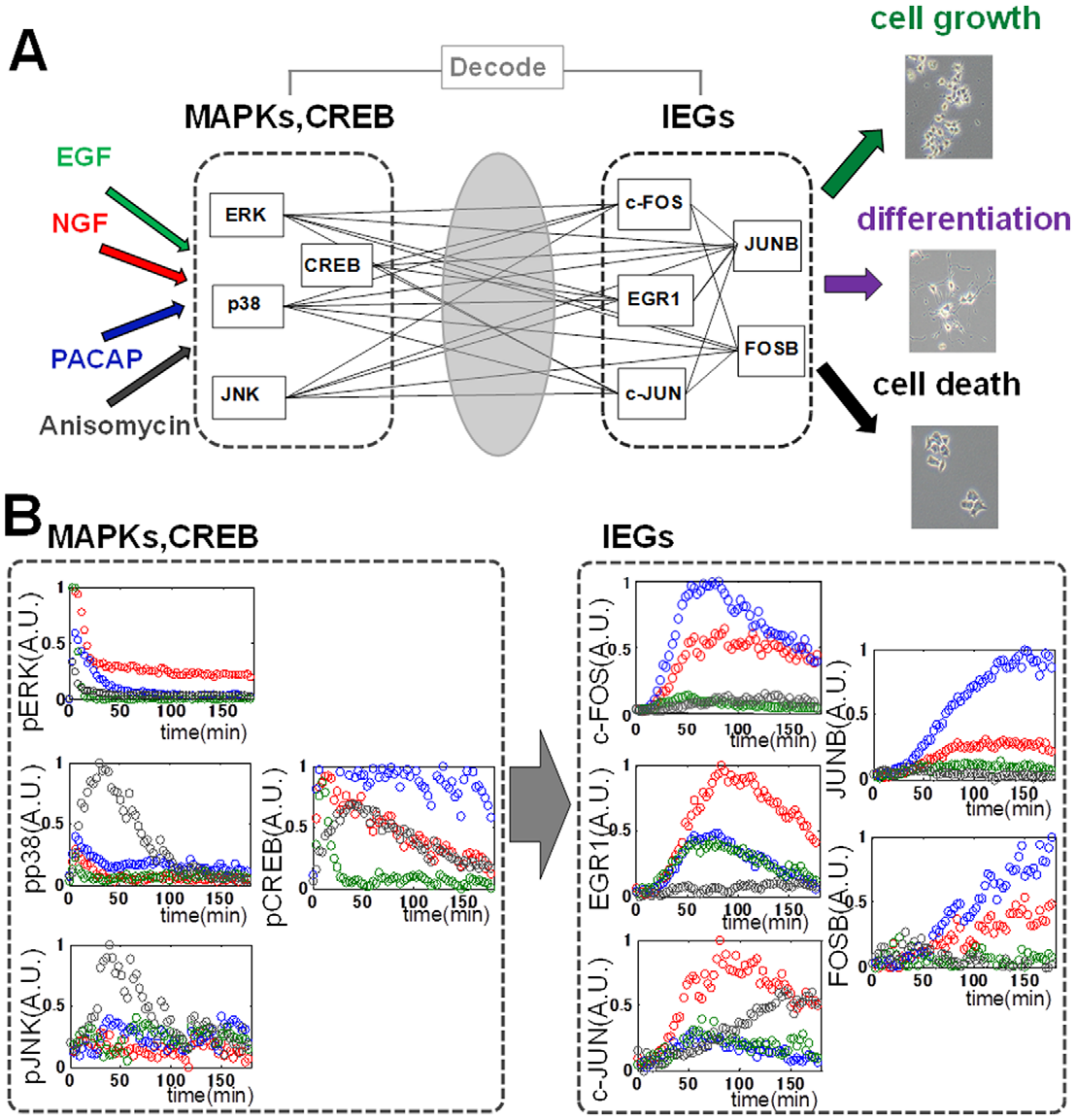
Decoding of MAPK and CREB phosphorylation by IEG expression. (A) A variety of growth factors such as EGF, NGF, PACAP, and anisomycin encode their information by specific temporal patterns of MAPK (ERK, p38, and JNK) and CREB phosphorylation, which are selectively decoded by expression of IEGs such as c-FOS, EGR1, c-JUN, JUNB, and FOSB to exert biological functions. (B) The temporal patterns of phosphorylation of MAPKs and CREB, and the expression of IEGs in response to NGF (5 ng/ml, red), PACAP (100 nM, blue), EGF (5 ng/ml, green), and anisomycin (50 ng/ml, black) were measured by QIC at 3-min intervals for 180 min. These data, together with responses to other doses of the growth factors (Figure S3), were used for parameter estimation of the nonlinear ARX model in Figure 2. Intensities of the signaling activity and the IEGs between experiments were normalized by internal control of each 96 well plate.

MAP kinases (MAPKs) and CREB and the immediate early gene products (IEGs) have been shown to comprise a core processor of cellular information with limited numbers of molecular species [1]–[3]. Many studies have been attempted to examine signaling specificity [4]–[6]. However, how a wide range of growth factors encode information into specific temporal patterns and combinations of signaling molecules such as MAPKs, including ERK, JNK, p38, and CREB, that are further decoded by expression of IEGs including c-FOS, EGR1, c-JUN, FOSB, and JUNB to exert biological functions, remains to be elucidated (Figure 1A) [7]–[9]. For example, nerve growth factor (NGF) has been shown to encode information for cell differentiation by sustained ERK phosphorylation, whereas epidermal growth factor (EGF) has been shown to encode information for cell proliferation into transient ERK phosphorylation in PC12 cells [9]–[12]. In contrast, pituitary adenylate cyclase – activating peptide (PACAP) has been shown to encode information for cell differentiation by ERK and CREB phosphorylation, the latter of which is mainly regulated by a cAMP-dependent pathway [13]. Anisomysin, a translation inhibitor, has been shown to encode information for cell death by JNK and p38 phosphorylation [14], [15]. Such specific temporal patterns and combinations of MAPK and CREB phosphorylation are further decoded by a limited numbers of IEGs to exert biological functions (Figure 1A). However, how such limited numbers of IEGs can selectively decode upstream signals remains unknown. Because the detailed biochemical network from MAPKs and CREB to the IEGs remains unknown, it is difficult to develop a computational model of biochemical networks based on the literature. Therefore, we employed a system identification method [16] that enabled us to build a data-driven model of the decoding system of MAPKs and CREB by IEG expression. The aim of system identification in this study is a quantitative, computational description of the input – output relationship from time courses of phosphorylated MAPKs (pMAPKs), phosphorylated CREB (pCREB), and IEG expression in response to various doses of different growth factors in order to determine how upstream signals are selectively decoded by downstream IEG expression.

Kinetic modeling based on biochemical reactions from the literature is often used for systems biological analysis of signaling pathway [17]–[19]. However, kinetic modeling explicitly uses biochemical reactions of known signaling pathways and requires the detailed knowledge of signaling pathway, which means that it is applicable only to the field with sufficient knowledge of signaling pathway. At the same time, this means that unknown pathway(s) is not modeled, and therefore, the model can not be able to capture the IO relationship for which the unknown pathway(s) is responsible. In contrast, data-driven modeling can identify system directly from experimental data without detailed knowledge of signaling pathway [17]–[19]. Therefore, the data-driven modeling can represent the IO relationship involving the unknown pathway(s). In particular, given that amplitude and temporal patterns of signaling activities are essential properties of cellular signaling, the dose response and time course of signaling activities characterize a cellular system. Therefore, we divided the characteristics of a cellular system into dose response and time course, and used data-driven model based on the time course data with doses of growth factors, and selected the nonlinear ARX model, which consist of amplitude conversion by Hill function and a linear temporal filter, as the data-driven modeling approach in this study. Regarding signaling pathways as transmission channel, the nonlinear ARX model directly gives an essential and inherent property of signal processing of the system without detailed knowledge of signaling pathways.

To build the data-driven model, a quantitative high-throughput measurement system for protein phosphorylation and protein expression are required. We have recently developed a fully automated assay technique, termed quantitative image cytometry (QIC) [20], which integrates a quantitative immunostaining technique and a high-precision image-processing algorithm for cell identification. QIC allows gathering huge amounts of quantitative data on protein phosphorylation and expression without personal skill variation. In this study, we used QIC to measure the time course of MAP kinases and CREB phosphorylation and expression the IEGs, and built the data-driven model to identify signal processing of the system. We found that specific temporal patterns and combinations of MAPKs and CREB phosphorylation can be decoded by selective IEG expression via distinct temporal filters and switch-like responses.

## Materials and Methods

### Antibodies

Mouse anti-phospho-ERK1/2 (Thr 202/Tyr 204) monoclonal antibody (mAb) (#9106), rabbit anti-phospho-CREB (Ser 133) mAb (#9198), rabbit anti-phopho-JNK (Thr183/Tyr185) mAb (#4668), rabbit anti-EGR1 mAb (#4154), rabbit anti-c-JUN mAb (#9165), rabbit anti-c-FOS mAb (#2250), rabbit anti-JUNB mAb (#3753) and rabbit anti-FOSB mAb (#2251) were purchased from Cell Signaling Technology (Beverly, MA). Rabbit anti-phospho p38 mAb (v1211) was purchased from Promega (Madison, WI).

### Cell culture and treatments

PC12 cells (kindly provided by Masato Nakafuku, Cincinnati Children's Hospital Medical Center, Ohio) [21] were cultured at 37°C under 5% CO_2_ in Dulbecco's modified Eagle's medium (DMEM) supplemented with 10% fetal bovine serum and 5% horse serum (Invitrogen, Carlsbad, CA), and stimulated by recombinant mouse β-NGF (R&D Systems, Minneapolis, MN), EGF (Roche, Mannheim, Germany), PACAP (Sigma, Zwijndrecht, The Netherlands), or anisomycin (EMD Biosciences, Inc., San Diego, CA) as previously described [21]. We used a low dose of anisomycin (50 nM) to activate p38 and JNK without inhibiting translation. For inhibitor experiment, we stimulated by NGF in the presence of 10 nM PD (PD0325901, a MEK inhibitor, Sigma Zwijndrecht, The Netherlands), 5 µM H89 (PKA inhibitor, Sigma Zwijndrecht, The Netherlands). The inhibitors were added 30 min before growth factor stimulation. For the QIC assays, cells were seeded at a density of 10^4^ cells per well in 96-well poly-L-lysine–coated glass-bottomed plates (Thermo Fisher Scientific, Pittsburgh, PA), and then starved in DMEM containing 25 mM HEPES and 0.1% bovine serum albumin for approximately 18 h before stimulation. Stimulations for cells seeded in 96-well microplates were performed by replacing the starvation medium with the medium containing the stimulant, using a liquid handling system (Biomek® NX Span-8, Beckman Coulter, Fullerton, CA) with an integrated heater-shaker (Variomag®, Daytona Beach, FL) and robotic incubator (STX-40, Liconic, Mauren, Liechtenstein). Note that all the cells within a plate were fixed simultaneously to prevent the exposure of the cells to formaldehyde vapor during the treatment.

### Quantitative Image Cytometry (QIC)

QIC was performed as previously described [20]. Briefly, after the stimulation of the growth factors, the cells were fixed, washed with phosphate-buffered saline (PBS), and permeabilised with blocking buffer (0.1% Triton X-100, 10% fetal bovine serum in PBS). The cells were washed and then incubated for 2 h with primary antibodies diluted in Can Get Signal immunostain Solution A (Toyobo, Osaka, Japan). The cells were washed three times and then incubated for 1 h with second antibodies. After immunostaining, the cells were stained for the nucleus and cytoplasm by incubating with Hoechst 33342 (Invitrogen) and CellMask Deep Red stain (Invitrogen), respectively. The images of the stained cells were acquired using the CellWoRx (Thermo Fisher Scientific) automated microscope with a ×10 objective. For QIC analyses, we acquired two different fields in each well, obtaining 1238±356 (mean ± SD) cells for each well. All liquid handling for the 96-well microplates was performed using a Biomek® NX Span-8 liquid handling system.

### System Identification by Nonlinear ARX model

We employed the nonlinear ARX model, which consisted of the linear ARX model [22] and variable transformation of inputs by Hill function [23]. The nonlinear ARX model is given by
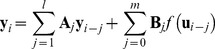
(1)where **A** *_j_* and **B** *_j_* are coefficient matrices of autoregressive and exogenous variables, respectively, **y** *_i_* and **u** *_i_* are the output vector of autoregressive variables and the input vector of exogenous variables, at discrete time *i*, respectively. *l* and *m* are the lag orders of autoregressive variables and exogenous variables, respectively. *f* is Hill function:



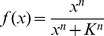
where *n* is the Hill coefficient and *K* is the dissociation constant that corresponds to the EC_50_, the half-maximal effective concentration of the input. However, the parameters of the Hill function does not always correspond to the meanings of biochemical reaction in this study. Note that, if we set *f*(*x*)  =  *x*, Equation (1) denotes the ordinary linear ARX model.

Coefficient matrices **A** *_j_* and **B** *_j_*, lag orders *l* and *m*, and the parameters of the Hill functions *n* and *K* were computationally determined by experimental data. Furthermore, molecular species; i.e., input variables were computationally selected. We used pERK, pJNK, pp38, and pCREB as the inputs for c-FOS, c-JUN, and EGR1, and pERK, pJNK, pp38, pCREB, c-FOS, c-JUN, and EGR1 for FOSB and JUNB for selecting the inputs based on earlier observations [24]. For simplicity, we consider that the lag orders *l* and *m* and the input variables are given. a) Coefficient matrices **A** *_j_* and **B** *_j_* were estimated to minimise sum of square error of one-step prediction by least square method for the fixed parameters of Hill functions *n* and *K*. b) The parameters of Hill functions *n* and *K* were estimated to minimise sum of square errors of one-step prediction for fixed coefficient matrices **A** *_j_* and **B** *_j_*. Each optimization step, a and b, was alternately iterated until the parameters converged (Figure S1). In practice, lag orders *l* and *m* and input variables were varied, and the optimization steps of parameters **A** *_j_*, **B** *_j_*, *n*, and *K* were done for each lag order *l* and *m* and input variables. We selected the model which had minimum of the average of Akaike Information Criteria (AIC) [25] of cross validation sets in varied lag orders *l* and *m* and input variables. In the optimization problem of estimating *n* and *K*, there were local minima. Therefore, 50 trials were done for the varied initial values of *n* and *K*.

## Results

### Systems identification of IEGs expression by MAP kinases and CREB

QIC enables us to measure phosphorylated ERK (pERK), phosphorylated JNK (pJNK), phosphorylated p38 (pp38), pCREB, and protein expression of the IEGs, including c-FOS, c-JUN, EGR1, JUNB, and FOSB, during 3-min intervals for 180 min in response to various growth factors such as NGF, PACAP, EGF, and anisomycin (Figure 1B, Figure S2 and S3). NGF, PACAP, EGF, and anisomycin induced distinct temporal patterns and combinations of pERK, pJNK, pp38, and pCREB and the IEGs (Figure 1B). This result suggests that the decoding system of the phosphorylation signals by the IEG expression is a multiple-input and multiple-output system, rather than a one-to-one correspondence system.

**Figure 2:**
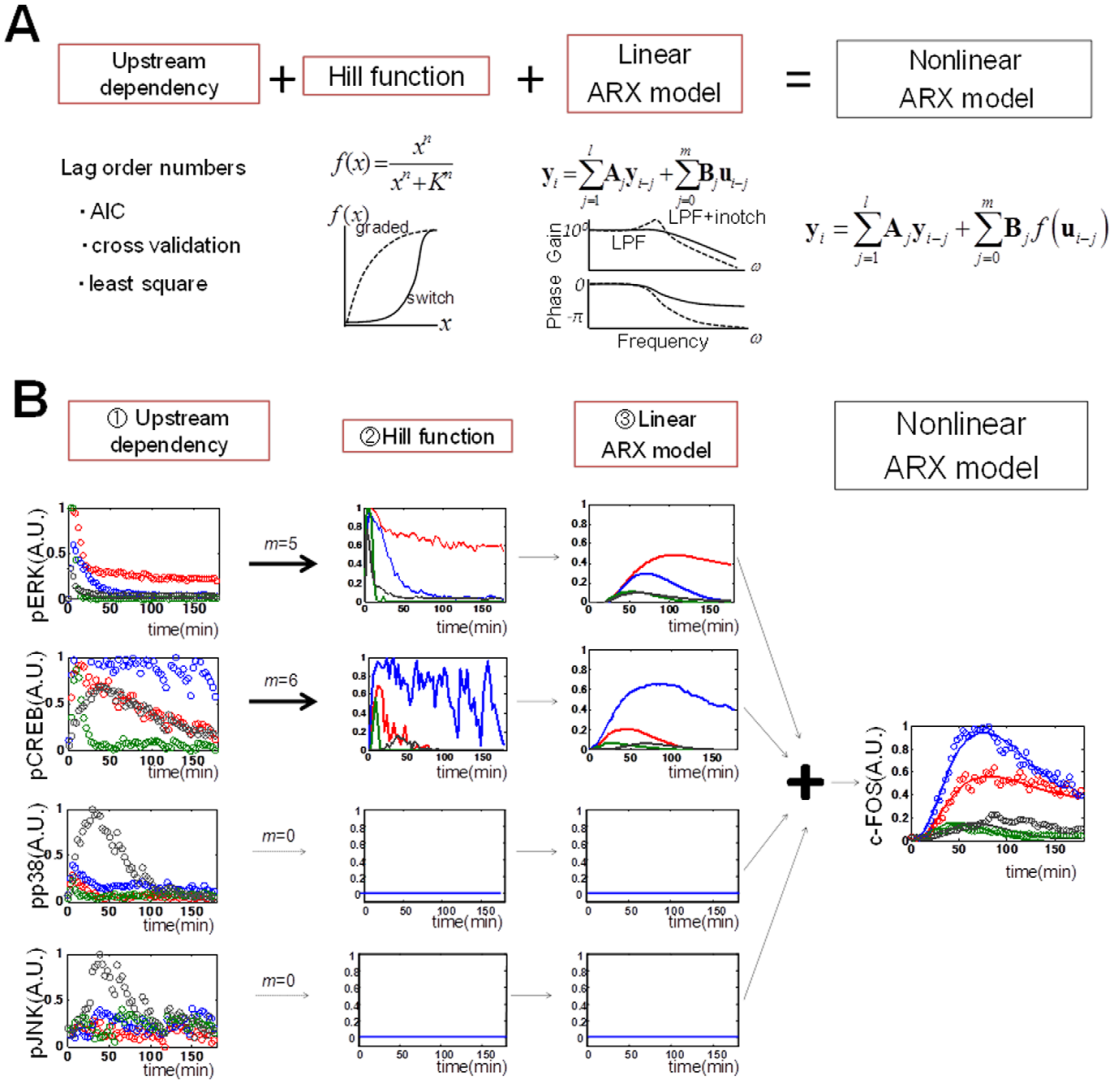
System identification by the nonlinear ARX model. (A) The modeling scheme of the nonlinear ARX model. Upstream dependency was determined by lag order number, *m*. For example, if *m* = 0, upstream signal is not transmitted downstream, otherwise signal is transmitted downstream. The signals of the selected upstream molecules were transformed successively by Hill function and linear ARX model, that characterise a system with switch-like (solid line) or graded (dotted line) dose response, and with temporal filters such as a low-pass filter (dotted line) and that with an inverse notch (solid line), respectively (see Materials and methods). (B) Temporal signal transformation in the nonlinear ARX model. For example, signal transformation in the nonlinear ARX model of c-FOS was shown. pERK and pCREB were selected upstream molecules, but pp38 and pJNK were not (*m* = 0). The signals of pERK and pCREB were transformed by the Hill equations. Then, the transformed signals by the Hill equations were temporally transformed by the linear ARX model. The sum of the transformed signals by the linear ARX model was c-FOS, the final output of the nonlinear ARX model of c-FOS.

From the measurement data (Figure 1B, Figure S3), we could build the data-driven model using a nonlinear autoregressive exogenous (ARX) model, in which the input signals are transformed successively by the Hill function and a linear temporal filter implemented by linear ARX model, a Hammerstein model [26] that works as a nonlinear temporal filter (Figure 2, see Materials and methods). The nonlinear ARX model characterises a system with upstream dependency, Hill functions, and linear ARX model. The upstream dependency was determined by lag order numbers, *m*, to the system according to a statistical criterion, Akaike Infomration Criterion (AIC) [25] (Table S1, see Materials and methods). For example, the signal of the molecule with *m*>0 is transmitted downstream, and that with *m* = 0 is not (Figure 2B). The Hill function transforms the amplitude of signals of the selected upstream molecules in a graded or switch-like manner, and the parameters of each Hill function were computationally tuned. Then, the transformed signals by the Hill function are temporally transformed by the linear ARX model. The sum of the transformed signals is the output of the nonlinear ARX model. The linear ARX model can represent linear temporal filter characteristics with a gain, a signal transfer efficiency in amplitude, and with a cutoff frequency, an inverse of time constant, and a phase shift. Note that the nonlinear ARX model is trivially equal to linear ARX model if Hill function is linear.

**Figure 3:**
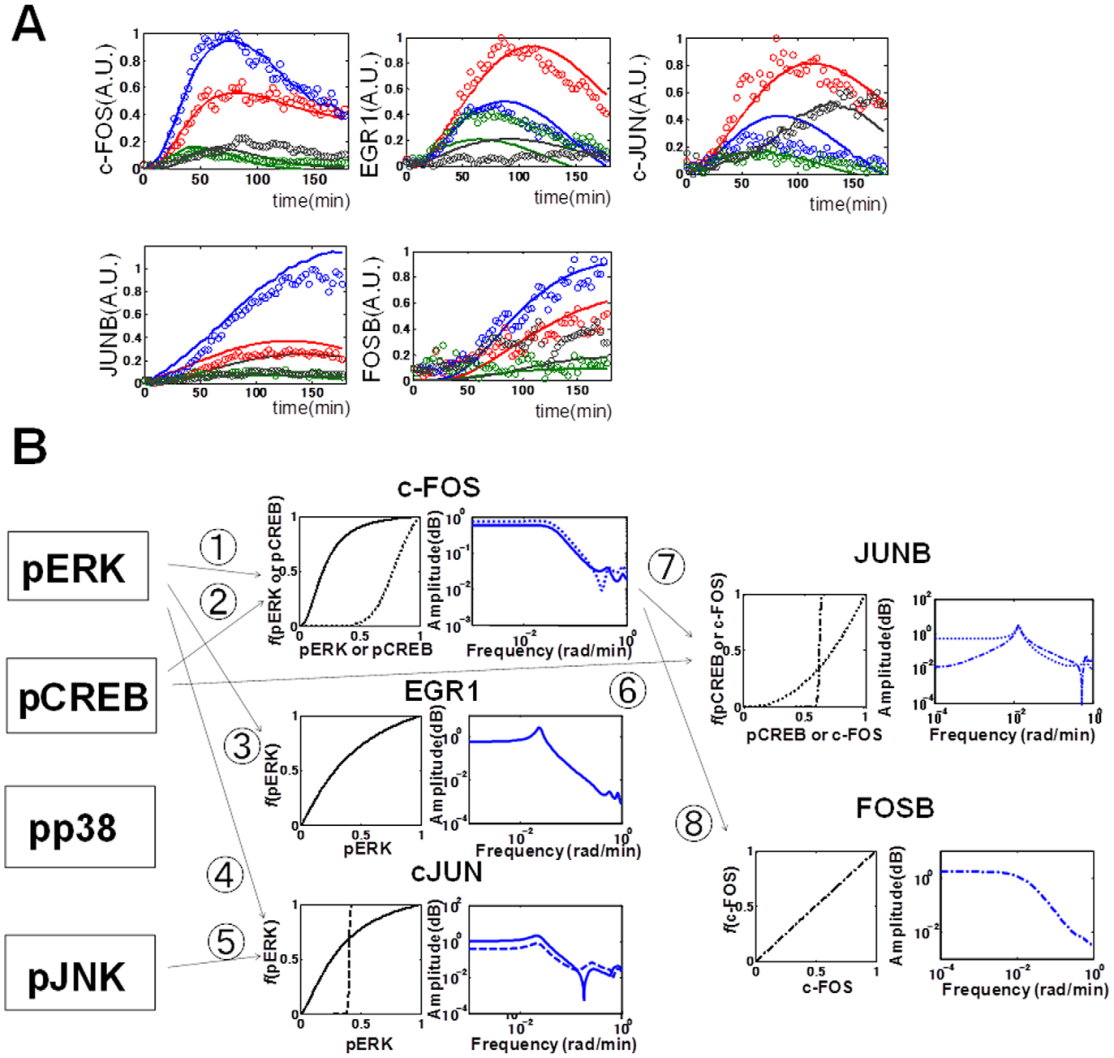
The nonlinear ARX model of the IEGs. (A) The simulation result of the nonlinear ARX model (solid lines) together with the experimental results in Figure 1B (dots). The colour codes are the same as in Figure 1B. The experimental data in Figure 1B and Figure S2 were used for parameter estimation of the nonlinear ARX model. (B) The identified systems by the nonlinear ARX model. The upstream dependency (selected inputs), Hill functions, and frequency response curve of the nonlinear ARX model were shown. The selected inputs, pERK (solid line), pCREB (dotted line), pJNK (dashed line), and c-FOS (dashed and dotted line) were numbered.

We applied the nonlinear ARX model to our experimental data (Figure 3A–B solid lines, Table 1, Figure S4 and S5, Table S1). The residual distribution of IEGs appeared similar to each other, and the mean and variation of each model were also similar (Figure S6), indicating that the nonlinear ARX model of each IEG could reasonably reproduce the experimental data. The time courses of the IEGs in the nonlinear ARX model equaled a linear sum of the time course of the previous input and output signals (Figure S5). In the nonlinear ARX model of c-FOS, pERK and pCREB were selected as the inputs for c-FOS expression (Figure 3B), which is consistent with previous observation [27]. To confirm the input dependency, we verified the model by using the inhibitors of signaling molecules, MEK inhibitor, PD0325901 and PKA inhibitor, H89 (Figure S7). The model reproduced experimental time course data, which means the input dependency derived from the model is reliable.

**Table 1:** The selected inputs and parameters of the Hill function and frequency response curves of the nonlinear ARX model.

Output	Pathway	Input	Hill function		Frequency response curve			
			K(EC50)	n	gain	filter characteristics.	cut off frequency (rad/min)	notch frequency (rad/min)
c-FOS	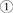	pERK	0.192	2.07	0.64	LPF	0.042	-
	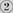	pCREB	0.776	7.683	0.81	LPF	0.044	-
EGR1	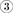	pERK	0.287	1.158	1.14	LPF + inotch	0.038	0.023
c-JUN	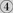	pERK	0.256	1.379	1.01	LPF	0.036	-
	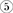	pJNK	0.400	100	0.38	LPF	0.036	-
JUNB	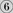	pCREB	0.732	2.24	0.52	LPF + inotch	0.021	0.013
	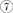	c-FOS	0.621	100	0.012	LPF + inotch	0.453	0.013
FOSB	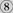	c-FOS	0.495	0.993	1.78	LPF	0.007	-

The Hill coefficients for pERK and pCREB were 2.070 and 7.683, respectively (Table 1). This indicates that signals of pCREB were transformed in switch-like manners (Figure 3B). The EC_50_s for pERK and pCREB were 0.192 and 0.776 (Table 1), respectively, indicating that pERK was more sensitive than pCREB at lower doses. The transformed input signals were then passed through the linear temporal filters (Figure 3B) and processed to the time series of the output, c-FOS (Figure 3A, Figure S5). The temporal filters resemble a low-pass filter for which the signal transfer efficiency at a lower frequency is better than that at a higher frequency. The cut-off frequencies of the filters for pERK and pCREB were 0.042 and 0.044 (radian/min), respectively, and the gains for pERK and pCREB were 0.64 and 0.81, respectively (Table 1). In the nonlinear ARX model of EGR1, EGR1 showed dependency only on pERK in a switch-like manner, and subsequent transformation by a low-pass – like temporal filter with an inverse notch (Figure 3B). In the nonlinear ARX models of c-JUN, FOSB and JUNB showed distinct upstream dependency, Hill functions, and linear temporal filter characteristics (Figure 3B, Table 1). Thus, expression of each IEG in response to a wide range of the inputs appeared as one or two inputs system rather than multiple-inputs system. The different characteristics between expression of each IEG highlights an ability for decoding the temporal patterns and combinations of the inputs, which suggests that expression of each IEG is regulated by distinct network structures. In addition, given that the single model for each IEG can reproduce the responses to all of the growth factors, this result suggests that the decoding of the pMAPKs and pCREB for expression of each IEG is the same regardless of growth factors, and that specificity for growth factors is generated at the level of the encoding step in temporal patterns and the combination of pMAPKs and pCREB. In the scheme of NARX model, the input-output relationship may include multi-step biochemical reactions. Therefore, the Hill coefficient of this model became higher than that of a single biochemical reaction. Furthermore, high Hill coefficient suggests a possible involvement of switch-like response, positive feedback loops or cooperativity [28].

### Temporal decoding of pulsatile ERK phosphorylation by the IEGs expression

**Figure 4:**
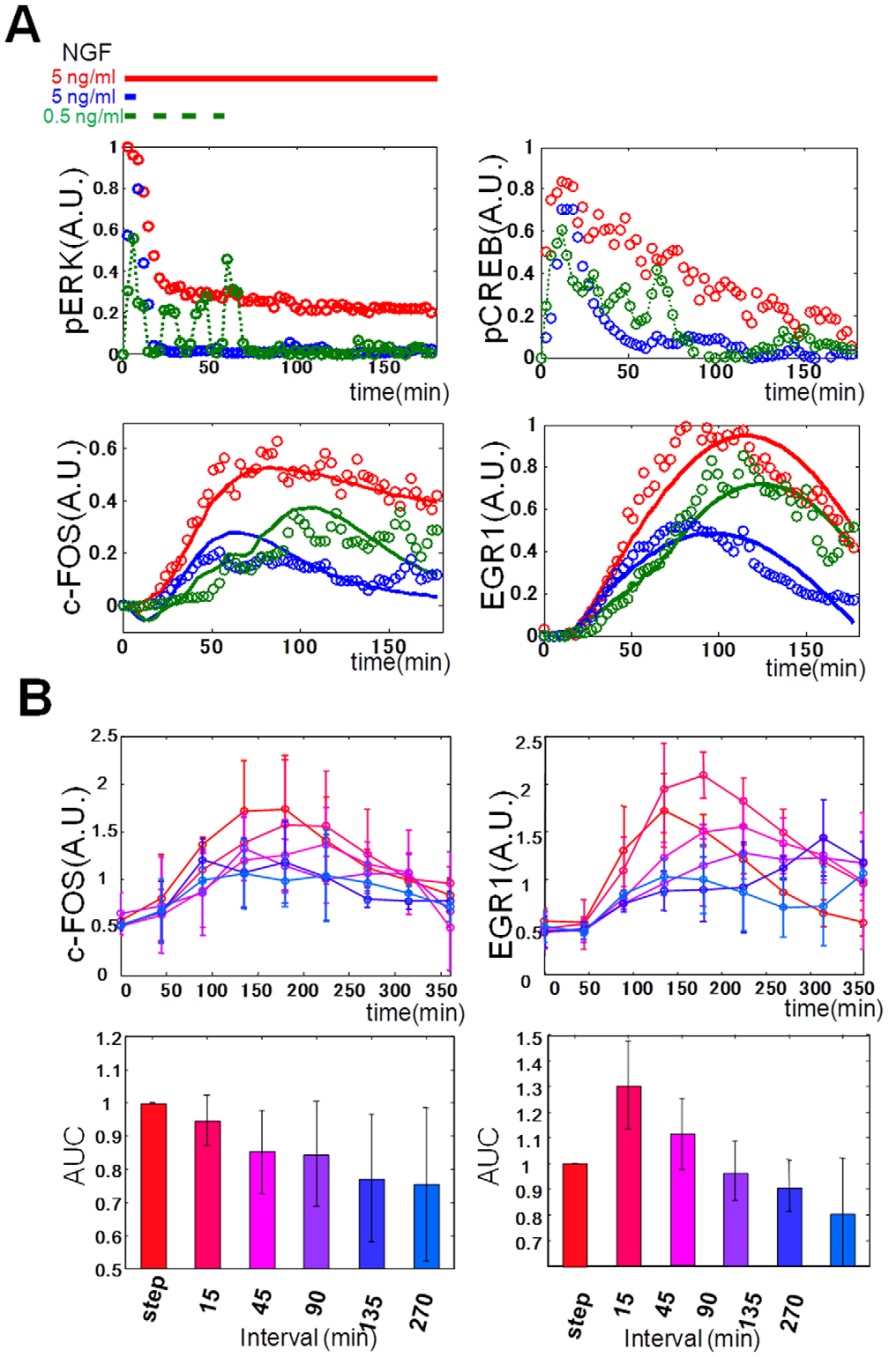
The selective expression of EGR1 in response to pulsatile ERK phosphorylation. (A) The step (5 ng/ml, red), pulse (5 ng/ml, 6 min, blue), and pulsatile NGF stimulation (0.5 ng/ml, 6 min with 12-min intervals for four times, green) were given as indicated by bars (top), and pERK, pCREB, EGR1, and c-FOS were measured in experiments (dots). Using the experimental data of pERK and pCREB as the selected inputs, the outputs (c-FOS and EGR1) were simulated by the nonlinear ARX model (solid lines). (B) Interval dependency of EGR1 and c-FOS expression. The pulsatile NGF stimulation (0.5 ng/ml, 15-min duration for each pulse) with the indicated intervals were given, and pERK, EGR1, and c-FOS expression were measured in experiments. The area under the curve (AUC) (0–480 min) of EGR1 and c-FOS are shown in bars. The intervals are indicated by the colour codes. Bars represent means ±S.D.(n = 4). Note that 15-min duration of pulses was used in Figure 4B because of the technical limitation of probe numbers of the automated liquid-handling robots, and pulsatile stimulation with 6-min pulse duration and 12-min intervals were available at most four times (Figure 4A).

To examine the validity of the model, we made an extrapolation (Figure 4). NGF has been shown to be secreted in a sustained [29] or pulse-like [30], [31] manner in vivo. This suggests that NGF secretion patterns induce different temporal patters of the MAPKs and the IEGs. Indeed, it has been reported that nuclear – cytoplasmic oscillation of ERK with a periodicity of 15 min was induced by EGF [32], and that oscillation of ERK activation is also induced by FGF [33], [34]. These findings led us to examine the possibility of selective decoding of temporal patterns of ERK phosphorylation by the IEG expression. Experimentally, we used the pulse and pulsatile stimulation of NGF and measured the molecules (Figure 4A, Figure S8) that were induced by NGF such as pERK, pCREB, EGR1, c-FOS, and c-JUN (Figure 1B). In experiments, the pulse and pulsatile stimulation of NGF induced transient and pulsatile pERK and pCREB (Figure 4A dots). The pulse and pulsatile stimulation of NGF induced expression of EGR1 more efficiently than c-FOS (Figure 4A dots, Figure S10), and the response of c-JUN was similar to that of c-FOS (Figure S8). Using the experimental data as input for the nonlinear ARX model, the model revealed that the pulse and pulsatile stimulation of NGF induced EGR1 expression more efficiently than c-FOS expression (Figure S5A, solid lines). There seem to be some differences between the simulation and experimental data for c-FOS. However, the experimental conditions such as temperature control and CO_2_ concentration was different from that of Fig. 1B. That may be one of the reasons why the simulated curves look different from those of experiments. Note that to control the stimulus environment is difficult even in the pulsatile stimulation. In the model, pERK was responsible for EGR1 (Figure 3), and pERK, but not pCREB, was also responsible for c-FOS expression (Figure S9). This indicates that the model can be valid for decoding the pulse and pulsatile patterns of pERK by the selective EGR1 expression. To examine whether the selective induction of EGR1 is because of the Hill function or the linear ARX model, we swapped the Hill function of pERK and the linear ARX models between EGR1 and c-FOS, and examined the response to a single pulse or pulsatile NGF stimulation (Figure S10). Swapping of either the Hill function or the linear ARX model resulted in similar response between the c-FOS and EGR1 models (Figure S10). This indicates that selective EGR1 expression depends on both the Hill function and the linear ARX model. We experimentally examined the effect of the interval of pulsatile NGF stimulation on EGR1 and c-FOS expression (Figure 4B), and on pERK and pCREB (Figure S11). EGR1 showed a maximal response to the pulsatile stimulation with a 15-min interval, and the response became smaller compared with those with longer intervals. On the other hand, c-FOS response was almost monotonically decreased as the interval of pulsatile stimulation became longer. Because the time points for measurements in Figure 3 and Figure 4B were different, the nonlinear ARX model in Figure 3 could not be directly applied to the data in Figure 4B. These results demonstrate that pulsatile ERK phosphorylation was selectively decoded by EGR1 expression.

### Decoding of conjunctive stimulation of NGF and PACAP by synergistic JUNB expression

**Figure 5:**
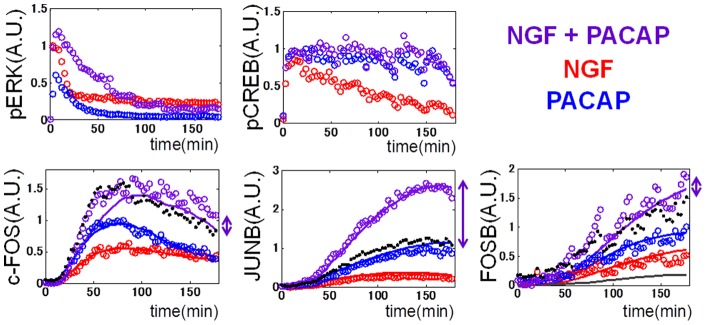
Conjunctive stimulation of NGF and PACAP induced synergistic JUNB expression through switch-like response to c-FOS. The step stimulation of NGF alone (5 ng/ml, red), PACAP alone (100 nM, blue), and both NGF and PACAP (violet) were given, and pERK, pCREB, c-FOS, JUNB, and FOSB were measured in experiments (dots). The simulation results of the nonlinear ARX model are shown (solid lines). Black dots indicate the sum of the IEG in response to NGF alone and to PACAP alone, and arrows indicate the difference from the sum.

We made another extrapolation of the model by using conjunctive stimulation of NGF and PACAP (Figure 5). NGF and PACAP have been reported to synergistically induce neurite extension [35], [36] and gene expression [37] in PC12 cells, and to simultaneously involve neural cell differentiation *in vivo* [38], [39]. Therefore, we examined whether the IEG expression could decode the conjunctive stimulation of NGF and PACAP. The conjunctive stimulation of NGF and PACAP induced higher intensity of pERK than NGF alone, while inducing pCREB to the same extent with PACAP alone (Figure 5 dots, Figure S12). Additive c-FOS and FOSB expression and synergistic JUNB expression were observed in both the experiment (Figure 5, dots) and the models (Figure 5 lines). The model revealed that synergistic JUNB expression is due to a switch-like response to c-FOS (Figure 3B, Table 1). Only c-FOS expression induced by NGF and PACAP – but not that induced by NGF or PACAP alone – exceeded the threshold of the switch-like responses. These results demonstrate that JUNB can serve as a selective detector of conjunctive stimulation of NGF and PACAP. As for the biological significance of selective detector of conjunctive stimulation, conjunctive stimulation of NGF and PACAP has been reported to induce different phenotypes like synergistic increase of ChAT mRNA [39] and neurite outgrowth [36].

## Discussion

### Temporal Coding for Biological Output

How the selective expression of IEGs is further decoded by the downstream genes to elicit specific biological output is the next central issue in temporal coding. For example, NGF or PACAP induced distinct combinations and temporal patterns and a combination of pMAPKs, pCREB, and IEGs (Figure 3); however, both growth factors similarly induced differentiation in PC12 cells [13]. This suggests that there may be common downstream decoders for cell differentiation. We have already identified *Metrnl*, *Dclk1*, and *Serpinb1a*, which are downstream genes of ERK [40] and essential for neurite extension in PC12 cells, as the common decoders of neurite length [41]. Expression levels of these genes, but not phosphorylation level of ERK, were always correlated with the neurite lengths in response to various doses of NGF or PACAP. Despite the distinct combinations and temporal patterns of the pMAPKs, pCREB, and the IEGs in response to NGF or PACAP (Figure 1B), the temporal expression patterns of these decoder genes were similar regardless of the growth factors. Taken together, these genes can decode information for neurite length that are encoded by distinct patterns of the pMAPKs, pCREB, and the IEGs. We will build the nonlinear ARX model using *Metrnl*, *Dclk1*, and *Serpinb1a* as the output and pMAPKs, pCREB, and IEGs as the inputs as a future project. This analysis will tell us how the distinct temporal patterns and combination of pMAPKs, pCREB, and IEGs in response to NGF or PACAP are decoded by *Metrnl*, *Dclk1*, and *Serpinb1a* expression for subsequent neurite extension. Similarly, biological outcomes and underlying expression of specific downstream genes in response to pulsatile NGF stimulation and conjunctive NGF and PACAP stimulation will be explored in the future.

It has been reported that nuclear – cytoplasmic oscillation of ERK with a periodicity of 15 min was induced by EGF [32]. Our finding suggests that such oscillation of ERK activation may also induce selective downstream gene expression under their conditions. The oscillation of ERK was observed at both microscopic single cell level and macroscopic cell population level [42]. In contrast, such oscillation of ERK (both phosphorylation and nuclear-cytoplasmic localization) was not observed at cell population level under our conditions (Fig. 1B). The fact that only step stimulation of NGF, but not the pulsatile NGF stimulation, induced c-FOS expression suggest that ERK was not oscillated in response to step stimulation of NGF, otherwise both types of NGF stimulation should similarly induce c-FOS expression. Therefore, NGF-induced oscillation of ERK does not likely to happen under our conditions, although we still can not exclude the possibility that NGF can induce oscillation of ERK at single cell level.

As for the possible physiological mechanism which enables EGR1 efficiently responsive to pulse and pulsatile stimulation of NGF, a negative feedback loop can be considered as a possible physiological mechanism responsive to pulse and pulsatile stimulation. Actually, NGF-dependent feedback loop through NAB2 has been reported [43]. We will plan to verify this possibility by experiment as a separate study.

### Possible Application Range of the Nonlinear ARX Model

The nonlinear ARX model could be applied in this study; however, because an ARX model is a linear model, the nonlinear ARX model may not be applicable to highly nonlinear behaviors such as bistability with hysteresis, which is mediated by a positive feedback loop, or relaxation oscillation, which is mediated by positive and delayed negative feedback loops such as bimodal distribution of MAP kinase phosphorylation [44], [45] or c-FOS expression [46]. Under our conditions, phosphorylation of MAPKs and expression of the IEG products always showed unimodal distributions, and apparent bistability was not observed [20]. Because of this, the nonlinear ARX model was capable of reproducing the averaged data in our study. Extension of the nonlinear ARX model by time-variant system will perform more appropriate model selection for the bistable systems, widening the application range of data-driven model for cellular functions.

**Figure 6:**
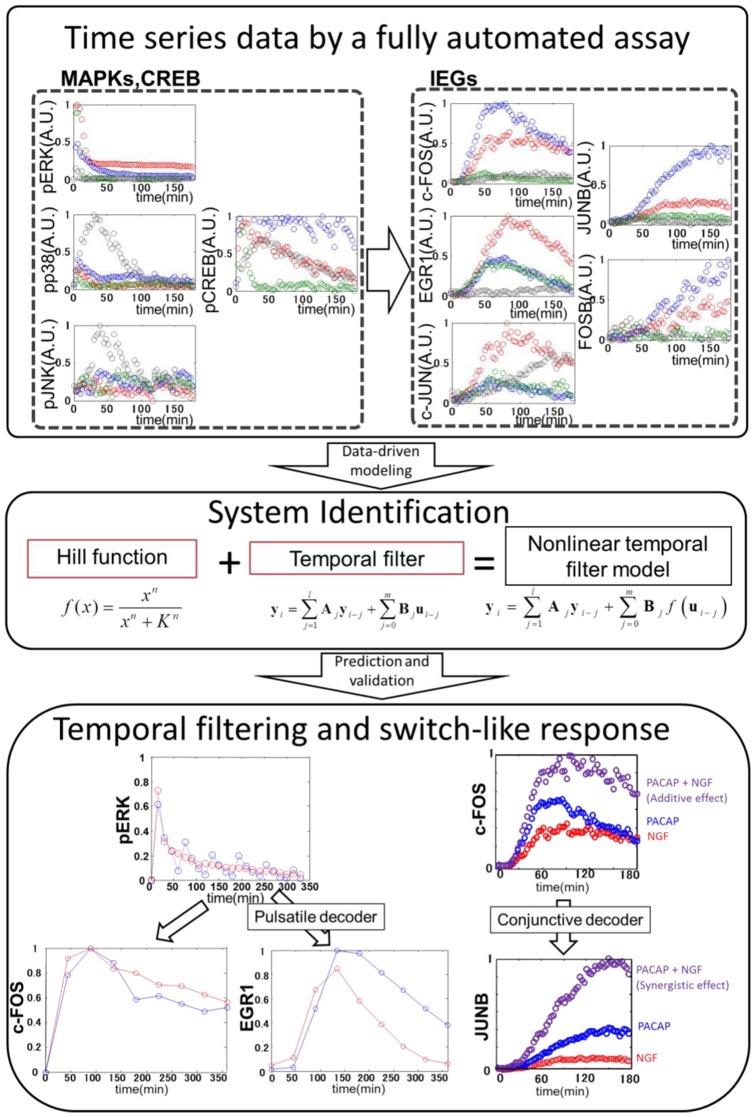
System identification reveals temporal decoding systems of MAP kinase and CREB phosphorylation by selective IEG expression. We made a system identification of temporal decoding of MAP kinase and CREB phosphorylation by selective immediate early genes expression such as c-FOS, EGR1, c-JUN and JUNB using time series data and the nonlinear ARX model. We found that the expression of IEGs has a distinct upstream dependency, and there are distinct switch-like responses and temporal filters for decoding upstream signals. For example, pulsatile ERK phosphorylation was decoded by selective expression of EGR1 rather than c-FOS, and conjunctive NGF and PACAP stimulation was decoded by synergistic JUNB expression through a switch-like response to c-FOS.

In conclusion, system identification revealed that temporal patterns and combinations of MAPK and CREB phosphorylation induced by a wide range of growth factors are decoded by the expression of selective IEGs via distinct upstream dependency, switch-like response, and linear temporal filter characteristics (Figure 6). We propose that such temporal and switch-like decoding is one of the design principles of cellular information processing. Furthermore, the system identification method provides a more quantitative and versatile approach than a conventional knowledge-based approach and can be used not only for cellular signaling and gene expression, but also for metabolic control and protein translation.

## Supporting Information

Figure S1 The illustration of parameter estimation procedure is shown. The model is selected by minimizing AIC with cross validation. The model structure is determined by the upstream dependency and the lag order. Note that the upstream dependency and lag order are discrete, hence we computed the model candidates and select the model which was the minimum of AIC with cross validation. The model parameters, which consist of parameters of Hill function and ARX, were estimated by the least square method for one-step prediction. The parameters of Hill function and ARX were alternately updated in the iterative procedure. If the difference of parameters between before updating and after updating was converged to approximately 0, the model parameters was almost at the local minima.
(TIF)

Figure S2 The relationship of the signal intensity of pCREB (left), FOSB (middle), and JUNB (right) between Western blotting (*x*-axis) and QIC (*y*-axis) are shown. Western blot images are also indicated below. The QIC for pCREB, FOSB, and JUNB show better sensitivity at lower intensity than western blotting.
(TIF)

Figure S3 The temporal patterns of pMAPKs and pCREB, and the expression of IEGs in response to NGF (0.5 ng/ml, magenta, 0.15 ng/ml, orange), PACAP (1 ng/ml, cyan), EGF (0.5 ng/ml, light green) are shown. Together with those in Figure 1B, these data were used for parameter estimation of the nonlinear ARX model in Figure 3.
(TIF)

Figure S4 The frequency response curve (Figure 3B) and phase plot of the linear ARX models for the indicated molecules in Figure 3 are shown. The selected inputs, pERK (solid line), pCREB (dotted line), pJNK (dashed line), and c-FOS (dashed and dotted line) are indicated.
(TIF)

Figure S5 The input signals in Figure 3 that were transformed successively by the Hill function and the summation of linear ARX model of c-FOS (A), c-JUN (B), and JUNB (C) in response to NGF (5 ng/ml, red, 0.5 ng/ml, magenta, 0.15 ng/ml, orange), PACAP (100 ng/ml, blue, 1 ng/ml, cyan), EGF (5 ng/ml, green, 0.5 ng/ml, light green) are shown. The output is composed of the linear sum of the transformed inputs.
(TIF)

Figure S6 The residual distribution of the IEGs expression (right) between experiment and simulation (left) in response to NGF (5 ng/ml, red, 0.5 ng/ml, magenta, 0.15 ng/ml, orange), PACAP (100 ng/ml, blue, 1 ng/ml, cyan), EGF (5 ng/ml, green, 0.5 ng/ml, light green)are shown. The mean (µ) and variation (σ^2^) of the residual distribution are also indicated.
(TIF)

Figure S7 The temporal patterns of pERK and pCREB, and the expression of IEGs in response to 5 ng/ml NGF in the presence of 10 nM PD (red dots) and 100 nM PACAP in the presence of 10 µM H89 (blue dots) were measured by QIC at 3-min interval. Using the experimental data of pERK, pCREB and c-FOS as the selected inputs, the outputs (c-FOS, EGR1, c-JUN, JUNB, FOSB) were simulated by the nonlinear ARX model (solid lines). Note that the temporal patterns of pERK, pCREB, and the IEGs in response to NGF (5 ng/ml, light red), PACAP (100 nM, light blue) are also shown (same experimental results in Figure 1B).
(TIF)

Figure S8 c-JUN expression in response to the step (red), a pulse (blue), and pulsatile NGF stimulation (green) are shown.
(TIF)

Figure S9 The inputs signals in Figure 4 that were transformed successively by Hill function and the summation of linear ARX model of c-FOS are shown. The linear sum of the c-FOS derived from pERK signals (left, top) and from pCREB signals (left, bottom) is c-FOS (right). The responses to step, pulse and pulsatile NGF stimulation are indicted by red, blue, and green, respectively.
(TIF)

Figure S10 Swapping of the Hill function of pERK or the linear ARX models between c-FOS and EGR1. (A) c-FOS and EGR1 expression in experiments (dots) and in simulation (lines) of the original nonlinear ARX model. The area under the curve (AUC) (0–480 min) in response to the step NGF stimulation for each model was set at 1, and the normalised area under the curves to a pulse and pulsatile NGF stimulation in experiment (exp.) and simulation (sim.) were indicated at the top of the bar. (B) The Hill functions of pERK or the linear ARX models between c-FOS and EGR1 were swapped as indicated, and the output responses are shown. The AUCs in response to a pulse and pulsatile NGF stimulation in the indicated swapped models became smaller than those in the original EGR1 model and larger than those in the original c-FOS model. This indicates that selective EGR1 expression depends on both the Hill function and the linear ARX model of EGR1.
(TIF)

Figure S11 Interval dependency of ERK and CREB phosphorylation. pERK and pCREB in response to the step (red) and pulsatile NGF stimulation with 15-min (blue) and 30-min (green) intervals are shown (top).
(TIF)

Figure S12 The selected inputs signals in Figure 5 that were transformed successively by Hill function and the summation of linear ARX model of c-FOS (A) and JUNB (B) are shown. The sum of the IEGs derived from the indicated inputs is the IEG. The responses to the step stimulation of NGF alone, PACAP alone and both NGF and PACAP are indicated by red, blue and violet, respectively.
(TIF)

Table S1 The parameters of the Hill functions and linear ARX model of each IEG are shown.
(DOC)
