# Supplementary figures and images for: Temporal Decoding of MAP Kinase and CREB Phosphorylation by Selective Immediate Early Gene Expression
Source: PLoS One. 2013 Mar 4;8(3):e57037. doi: 10.1371/journal.pone.0057037 (PMC3587639; doi:10.1371/journal.pone.0057037)

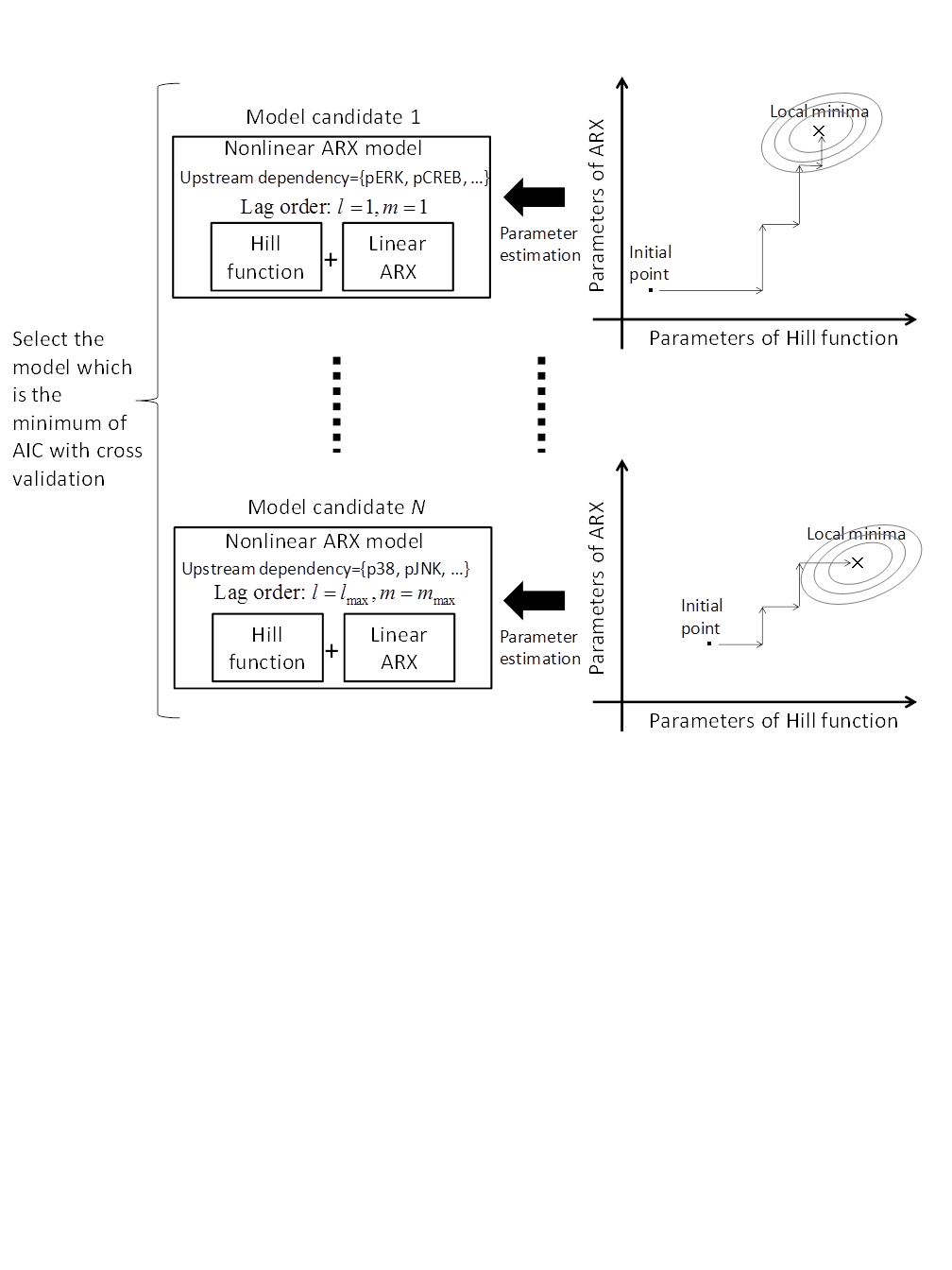

Supplement: Figure S1 — The illustration of parameter estimation procedure is shown. The model is selected by minimizing AIC with cross validation. The model structure is determined by the upstream dependency and the lag order. Note that the upstream dependency and lag order are discrete, hence we computed the model candidates and select the model which was the minimum of AIC with cross validation. The model parameters, which consist of parameters of Hill function and ARX, were estimated by the least square method for one-step prediction. The parameters of Hill function and ARX were alternately updated in the iterative procedure. If the difference of parameters between before updating and after updating was converged to approximately 0, the model parameters was almost at the local minima. (TIF) [file pone.0057037.s001.tif]

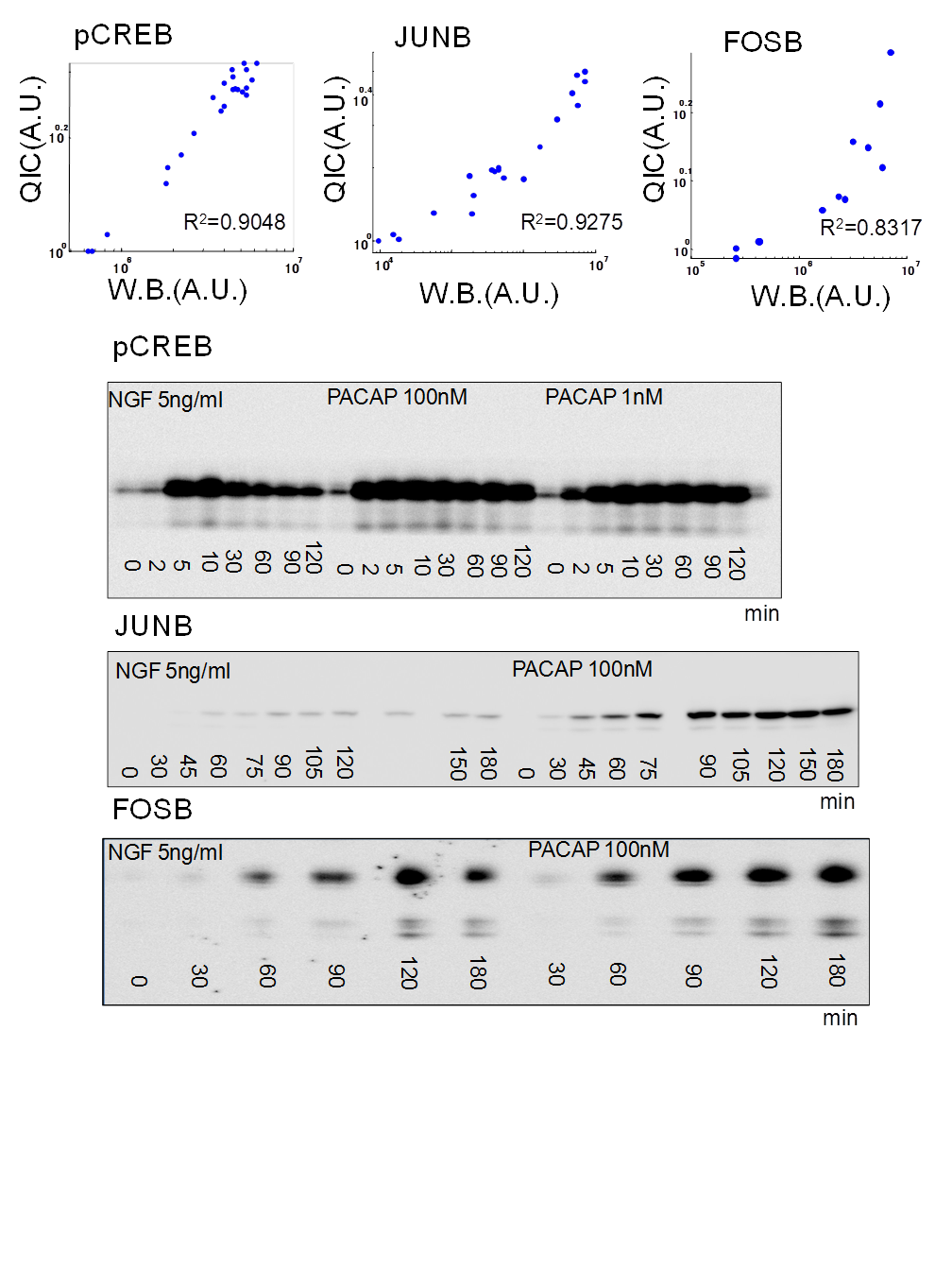

Supplement: Figure S2 — The relationship of the signal intensity of pCREB (left), FOSB (middle), and JUNB (right) between Western blotting (x-axis) and QIC (y-axis) are shown. Western blot images are also indicated below. The QIC for pCREB, FOSB, and JUNB show better sensitivity at lower intensity than western blotting. (TIF) [file pone.0057037.s002.tif]

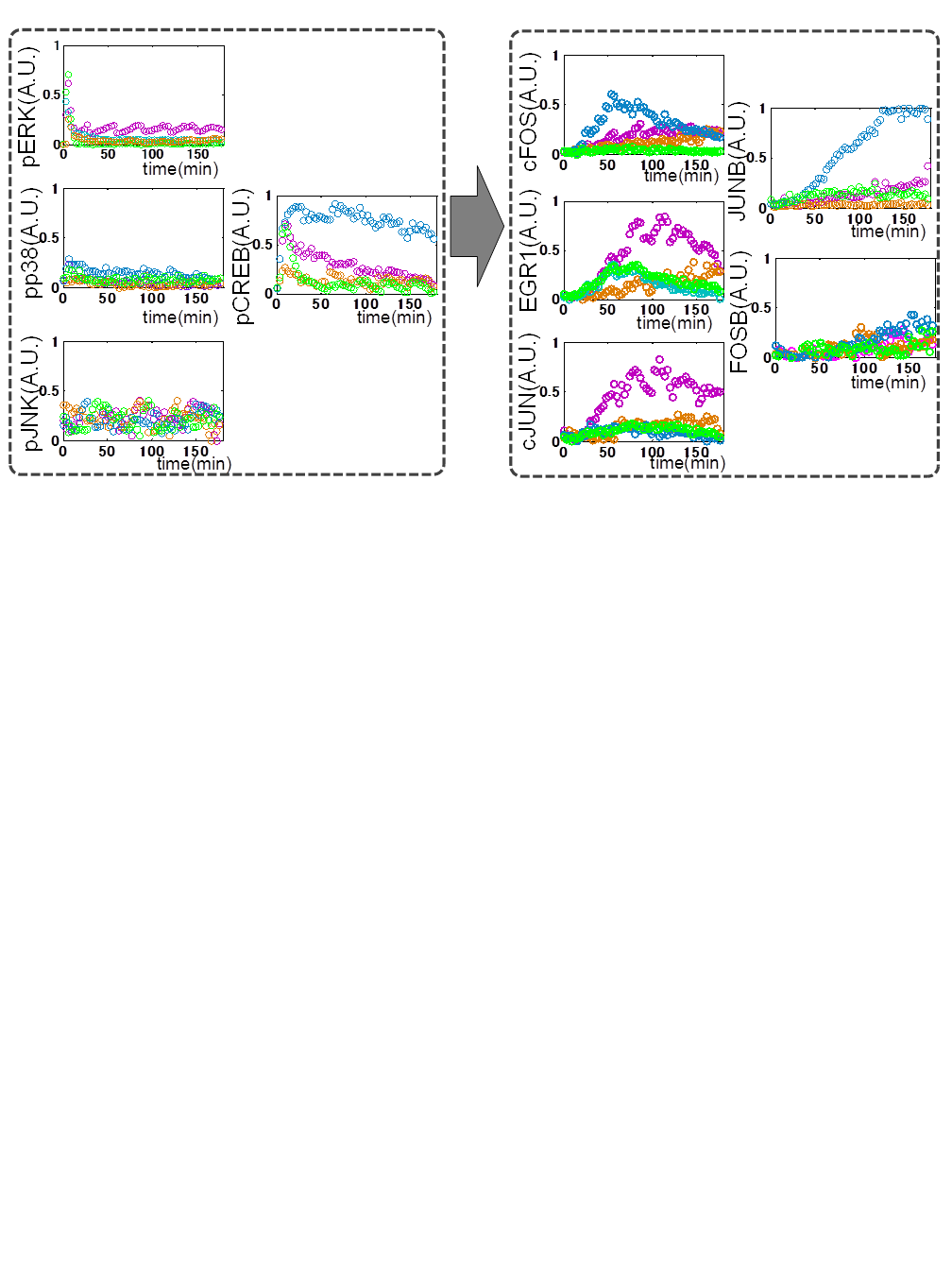

Supplement: Figure S3 — The temporal patterns of pMAPKs and pCREB, and the expression of IEGs in response to NGF (0.5 ng/ml, magenta, 0.15 ng/ml, orange), PACAP (1 ng/ml, cyan), EGF (0.5 ng/ml, light green) are shown. Together with those in Figure 1B, these data were used for parameter estimation of the nonlinear ARX model in Figure 3. (TIF) [file pone.0057037.s003.tif]

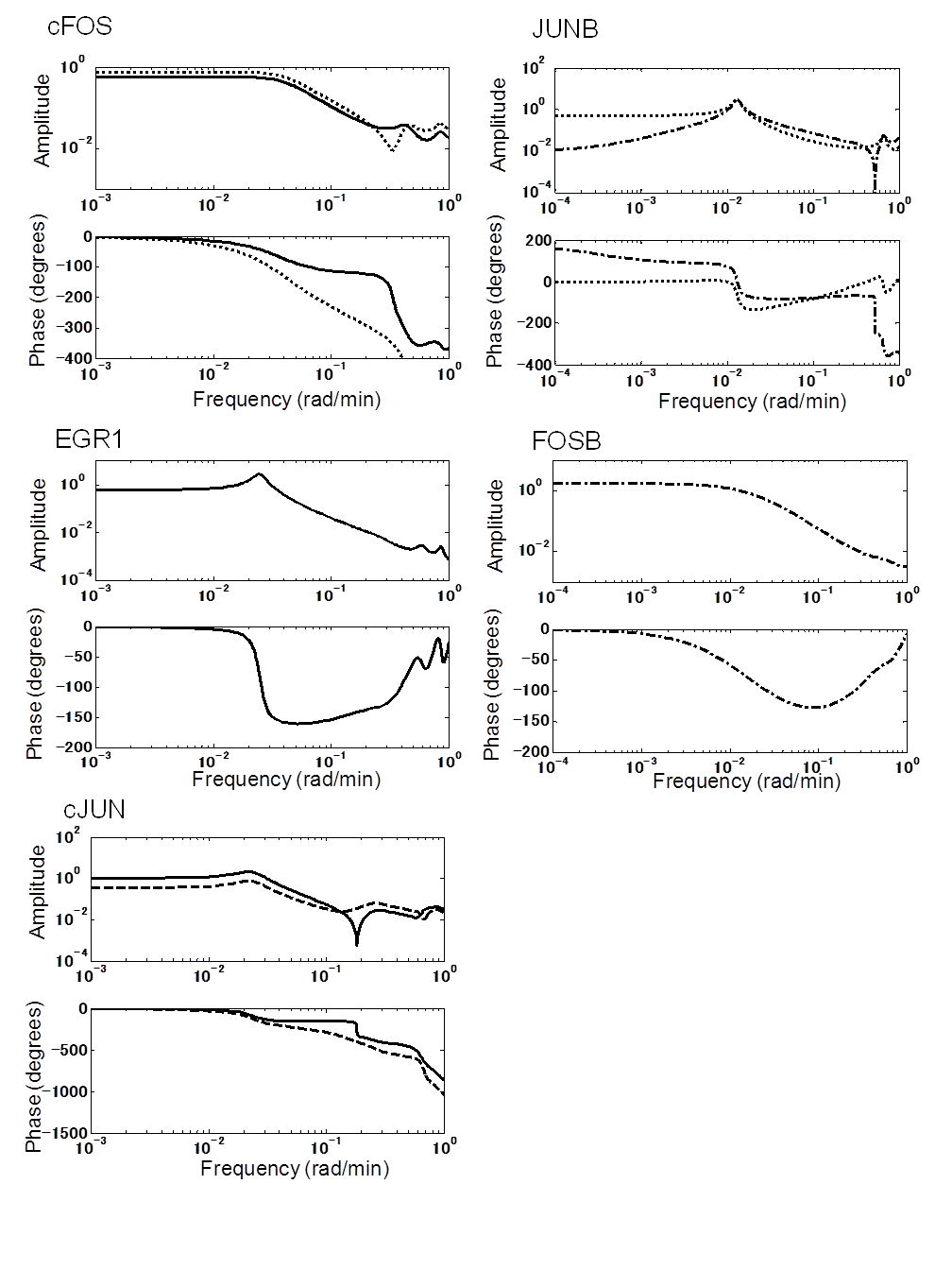

Supplement: Figure S4 — The frequency response curve (Figure 3B) and phase plot of the linear ARX models for the indicated molecules in Figure 3 are shown. The selected inputs, pERK (solid line), pCREB (dotted line), pJNK (dashed line), and c-FOS (dashed and dotted line) are indicated. (TIF) [file pone.0057037.s004.tif]

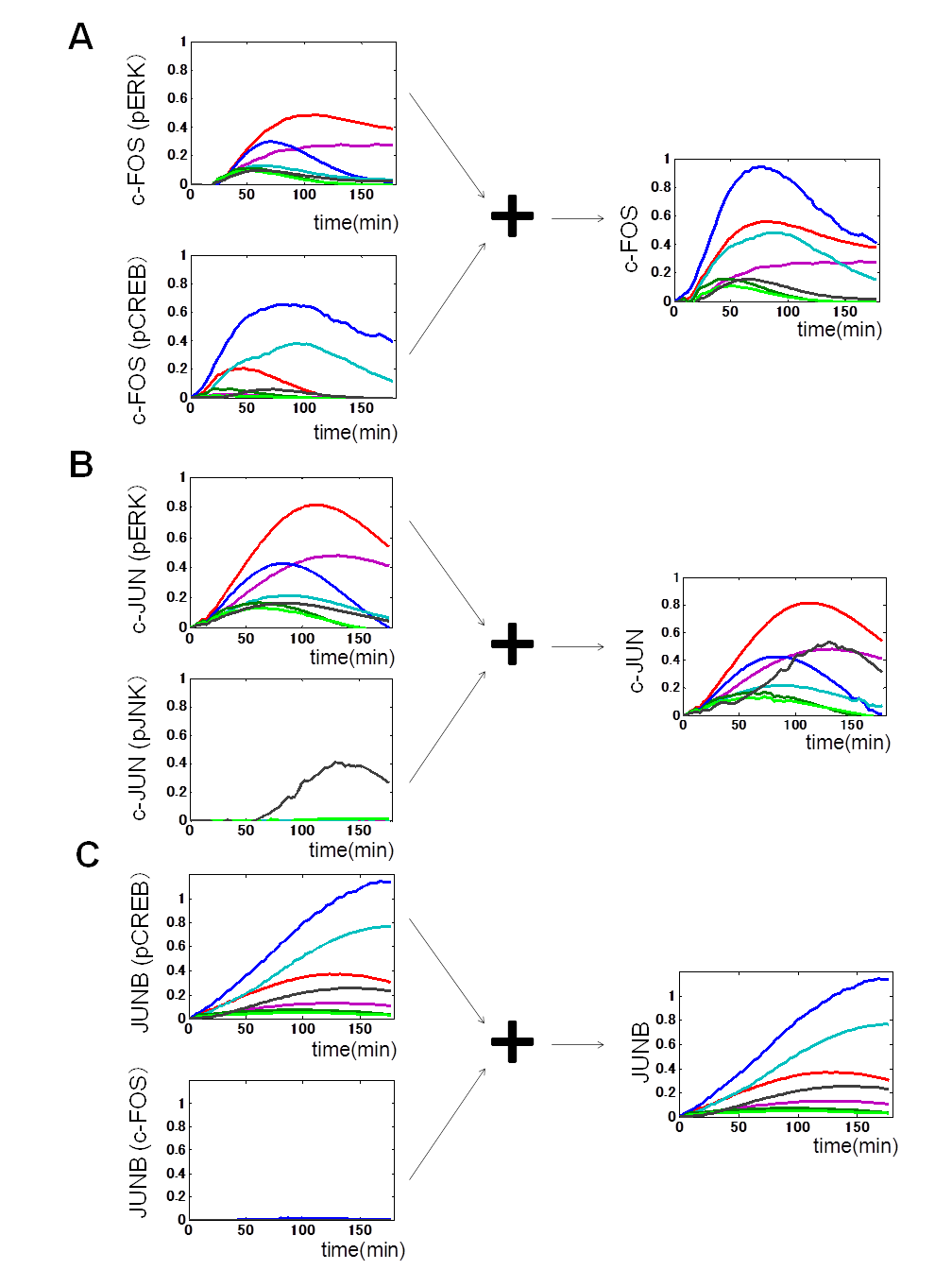

Supplement: Figure S5 — The input signals in Figure 3 that were transformed successively by the Hill function and the summation of linear ARX model of c-FOS (A), c-JUN (B), and JUNB (C) in response to NGF (5 ng/ml, red, 0.5 ng/ml, magenta, 0.15 ng/ml, orange), PACAP (100 ng/ml, blue, 1 ng/ml, cyan), EGF (5 ng/ml, green, 0.5 ng/ml, light green) are shown. The output is composed of the linear sum of the transformed inputs. (TIF) [file pone.0057037.s005.tif]

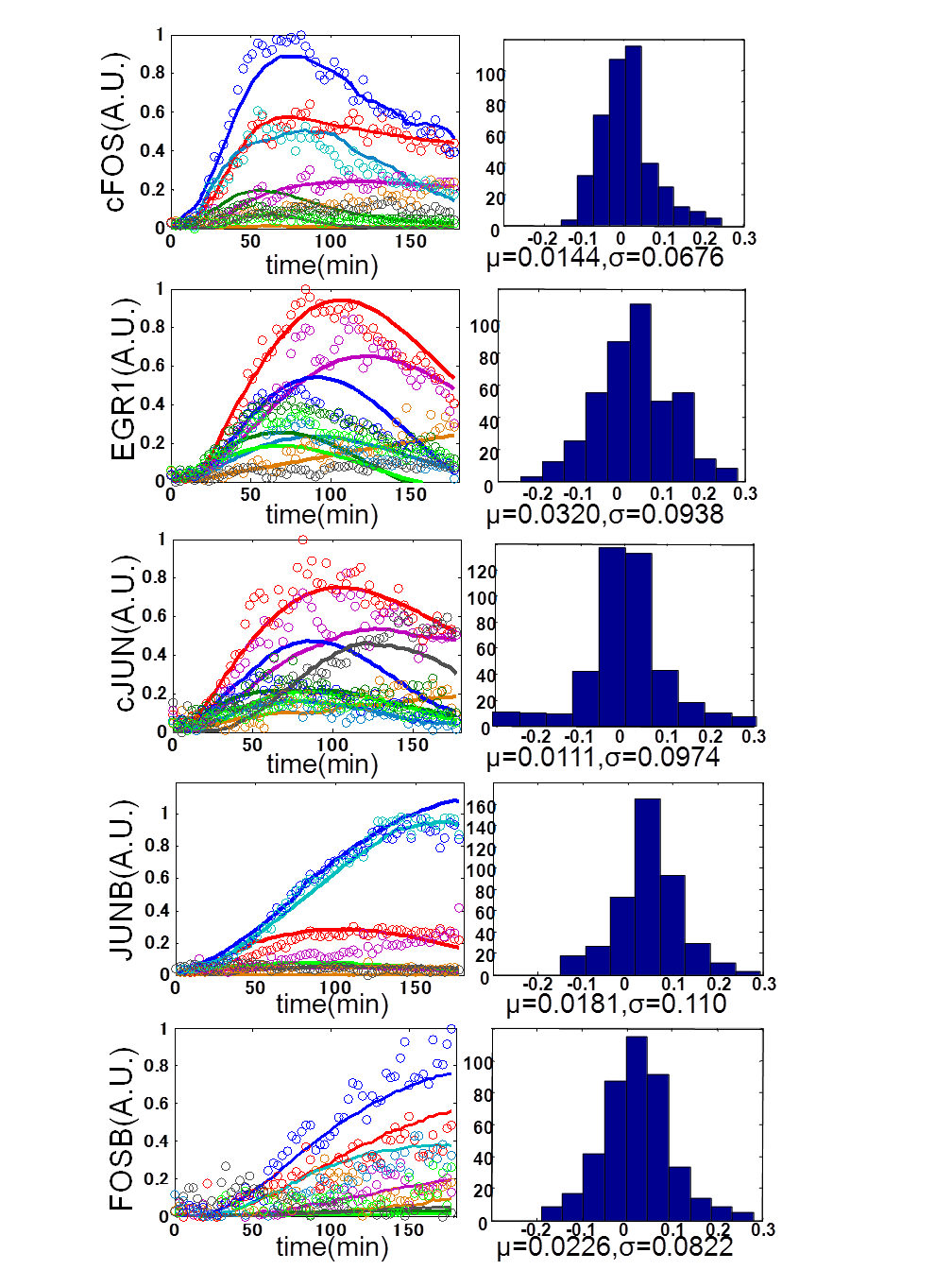

Supplement: Figure S6 — The residual distribution of the IEGs expression (right) between experiment and simulation (left) in response to NGF (5 ng/ml, red, 0.5 ng/ml, magenta, 0.15 ng/ml, orange), PACAP (100 ng/ml, blue, 1 ng/ml, cyan), EGF (5 ng/ml, green, 0.5 ng/ml, light green)are shown. The mean (µ) and variation (σ2) of the residual distribution are also indicated. (TIF) [file pone.0057037.s006.tif]

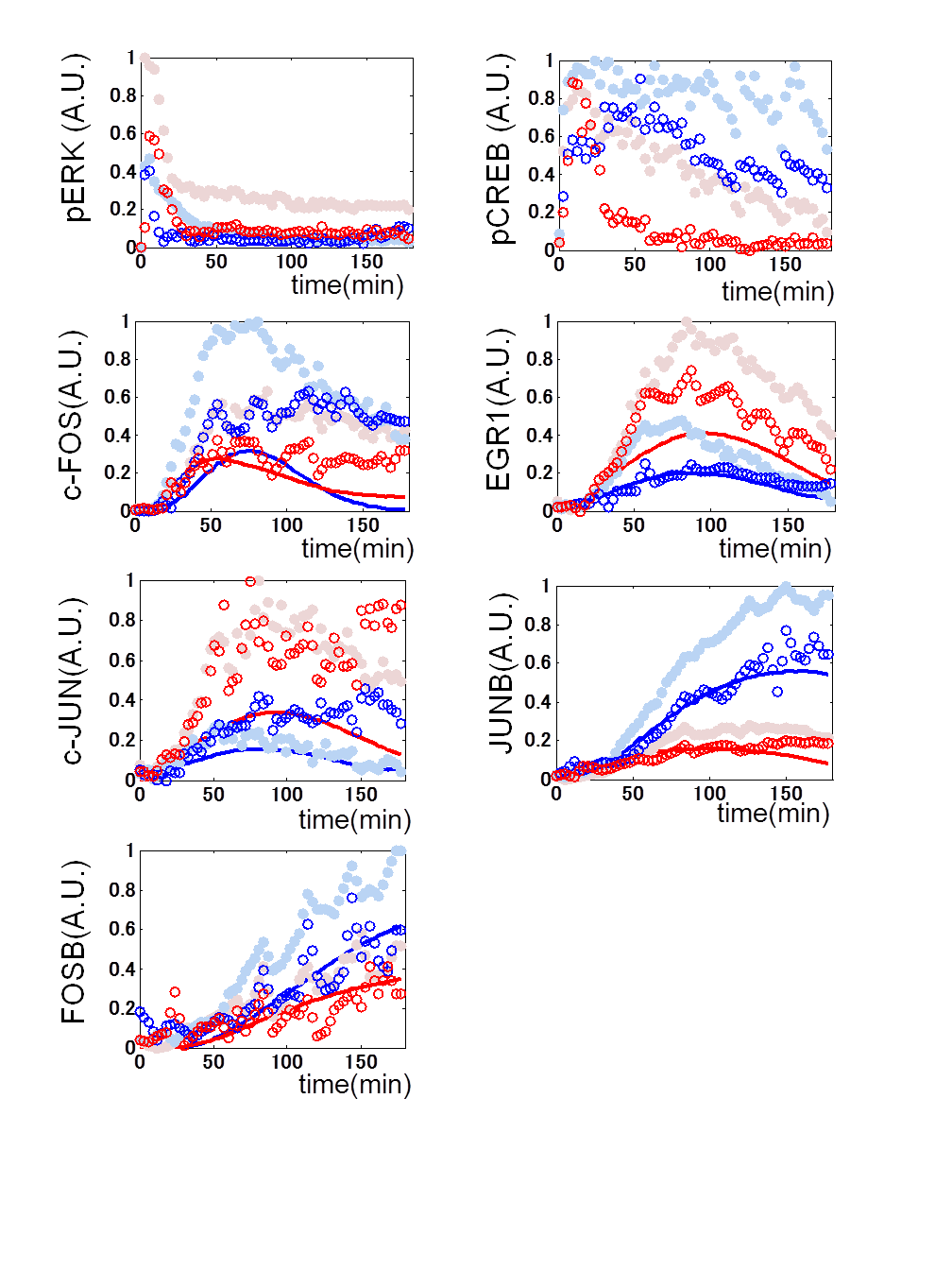

Supplement: Figure S7 — The temporal patterns of pERK and pCREB, and the expression of IEGs in response to 5 ng/ml NGF in the presence of 10 nM PD (red dots) and 100 nM PACAP in the presence of 10 µM H89 (blue dots) were measured by QIC at 3-min interval. Using the experimental data of pERK, pCREB and c-FOS as the selected inputs, the outputs (c-FOS, EGR1, c-JUN, JUNB, FOSB) were simulated by the nonlinear ARX model (solid lines). Note that the temporal patterns of pERK, pCREB, and the IEGs in response to NGF (5 ng/ml, light red), PACAP (100 nM, light blue) are also shown (same experimental results in Figure 1B). (TIF) [file pone.0057037.s007.tif]

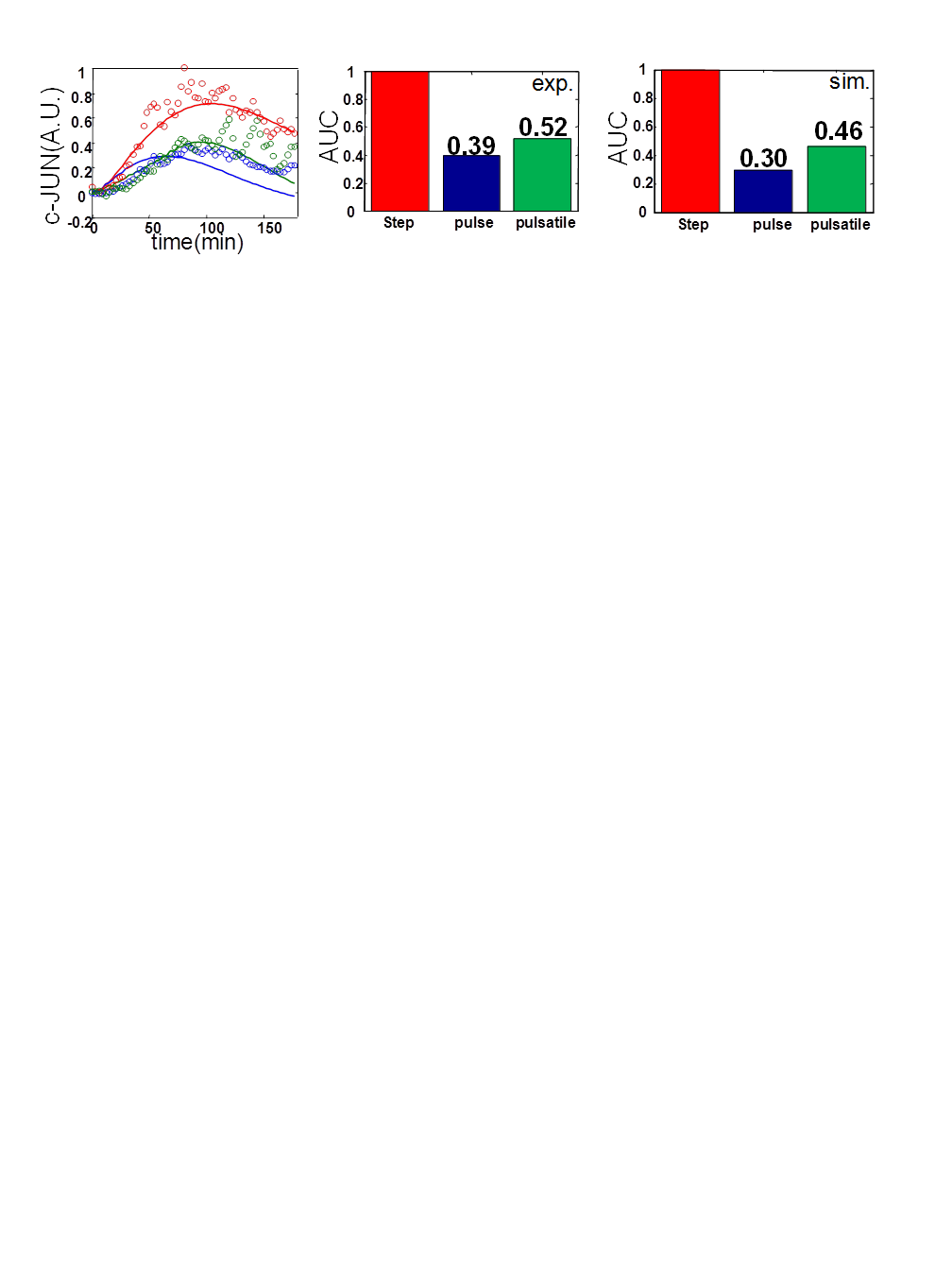

Supplement: Figure S8 — c-JUN expression in response to the step (red), a pulse (blue), and pulsatile NGF stimulation (green) are shown. (TIF) [file pone.0057037.s008.tif]

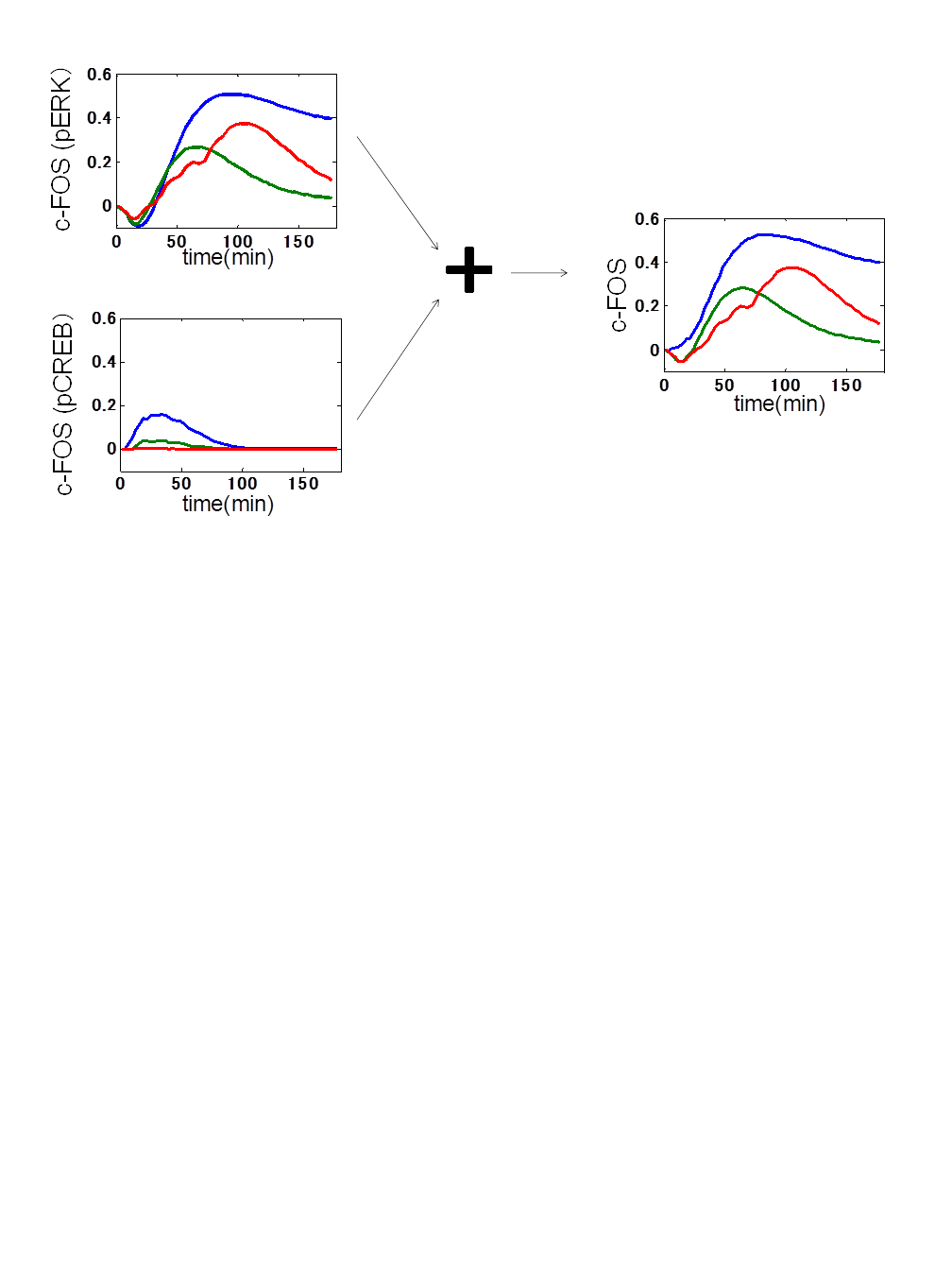

Supplement: Figure S9 — The inputs signals in Figure 4 that were transformed successively by Hill function and the summation of linear ARX model of c-FOS are shown. The linear sum of the c-FOS derived from pERK signals (left, top) and from pCREB signals (left, bottom) is c-FOS (right). The responses to step, pulse and pulsatile NGF stimulation are indicted by red, blue, and green, respectively. (TIF) [file pone.0057037.s009.tif]

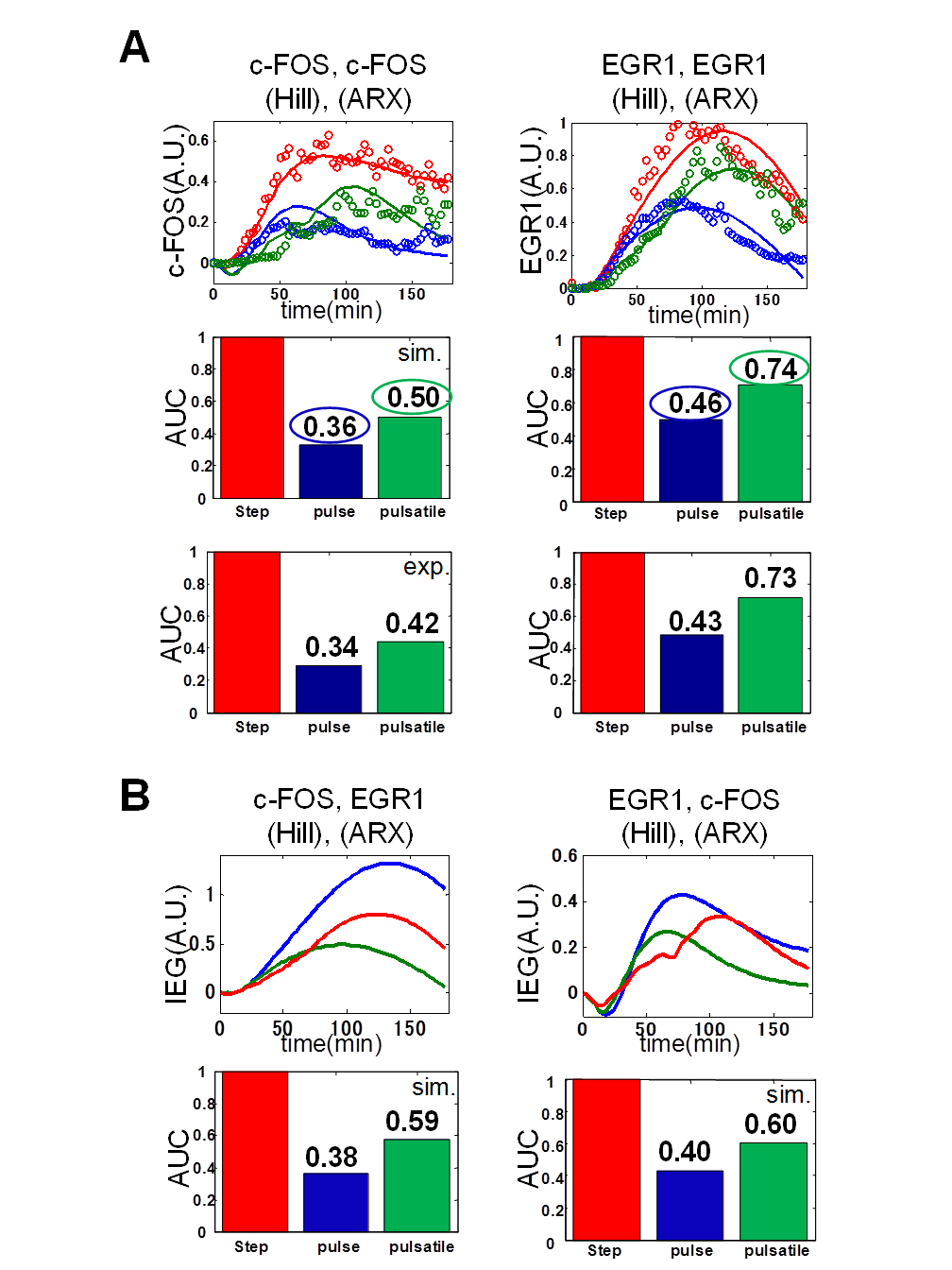

Supplement: Figure S10 — Swapping of the Hill function of pERK or the linear ARX models between c-FOS and EGR1. (A) c-FOS and EGR1 expression in experiments (dots) and in simulation (lines) of the original nonlinear ARX model. The area under the curve (AUC) (0–480 min) in response to the step NGF stimulation for each model was set at 1, and the normalised area under the curves to a pulse and pulsatile NGF stimulation in experiment (exp.) and simulation (sim.) were indicated at the top of the bar. (B) The Hill functions of pERK or the linear ARX models between c-FOS and EGR1 were swapped as indicated, and the output responses are shown. The AUCs in response to a pulse and pulsatile NGF stimulation in the indicated swapped models became smaller than those in the original EGR1 model and larger than those in the original c-FOS model. This indicates that selective EGR1 expression depends on both the Hill function and the linear ARX model of EGR1. (TIF) [file pone.0057037.s010.tif]

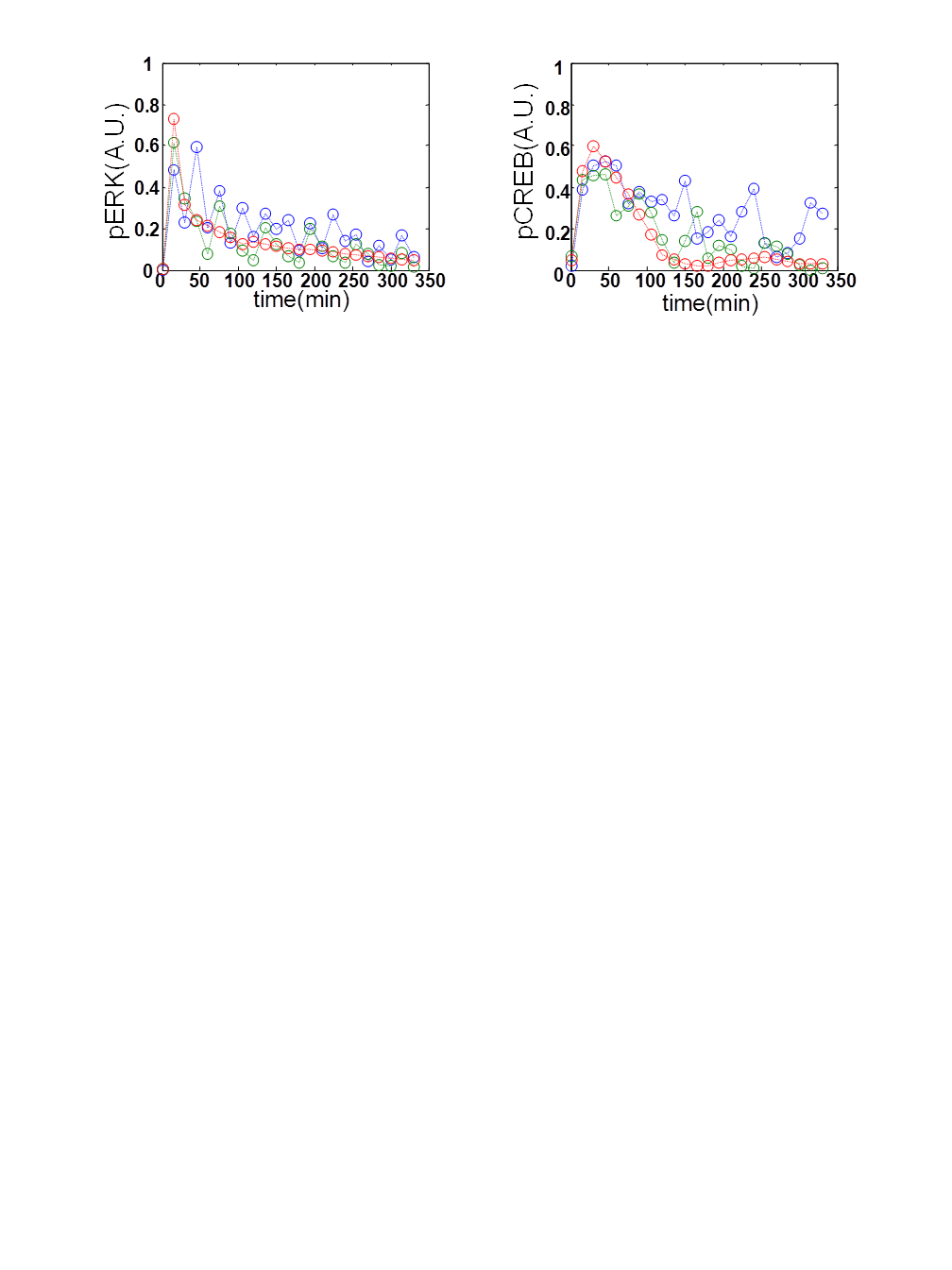

Supplement: Figure S11 — Interval dependency of ERK and CREB phosphorylation. pERK and pCREB in response to the step (red) and pulsatile NGF stimulation with 15-min (blue) and 30-min (green) intervals are shown (top). (TIF) [file pone.0057037.s011.tif]

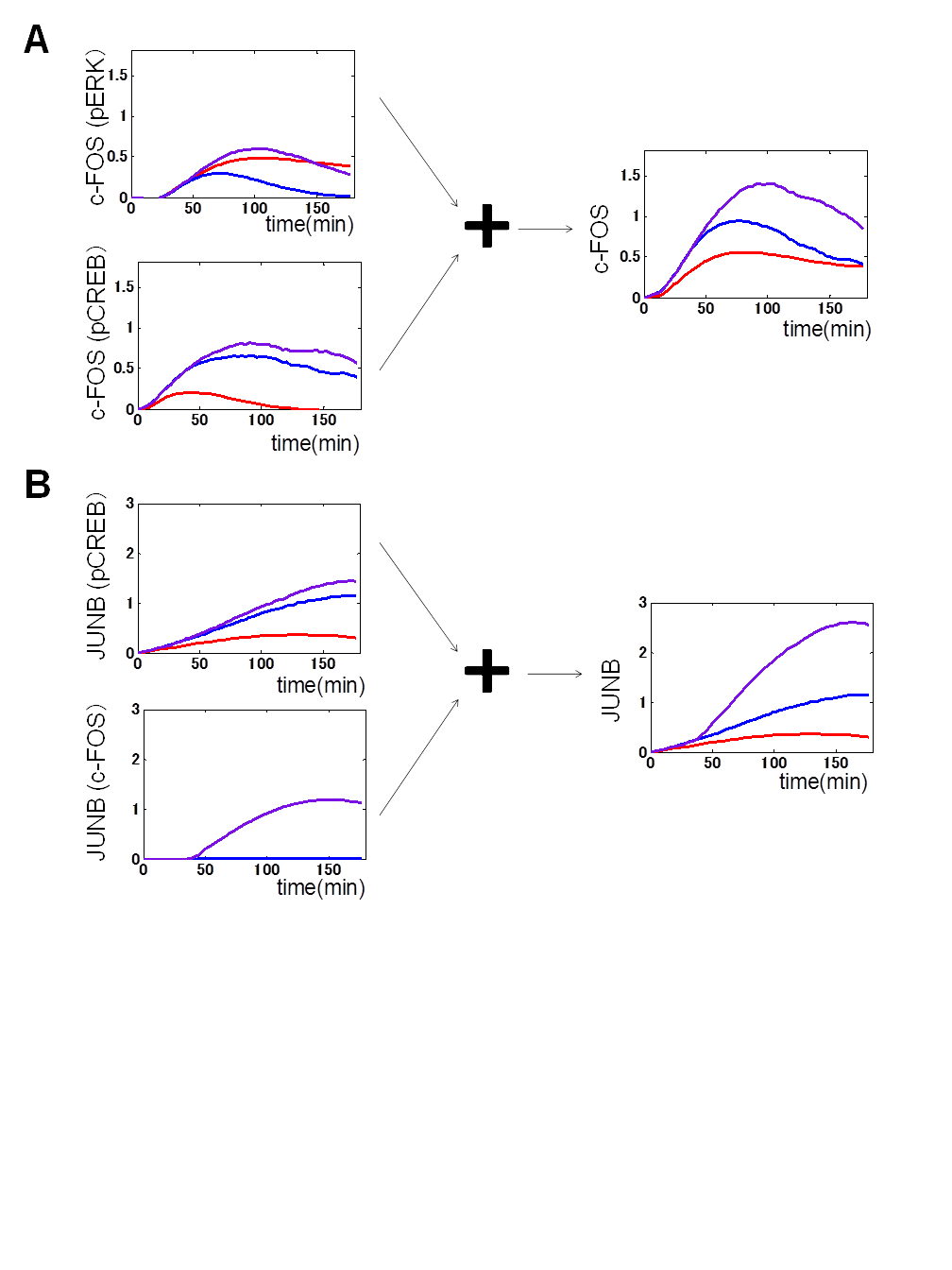

Supplement: Figure S12 — The selected inputs signals in Figure 5 that were transformed successively by Hill function and the summation of linear ARX model of c-FOS (A) and JUNB (B) are shown. The sum of the IEGs derived from the indicated inputs is the IEG. The responses to the step stimulation of NGF alone, PACAP alone and both NGF and PACAP are indicated by red, blue and violet, respectively. (TIF) [file pone.0057037.s012.tif]
