# Supplementary material for: Temporal Decoding of MAP Kinase and CREB Phosphorylation by Selective Immediate Early Gene Expression
Source: PLoS One. 2013 Mar 4;8(3):e57037. doi: 10.1371/journal.pone.0057037 (PMC3587639; doi:10.1371/journal.pone.0057037)
Supplement: Table S1 — The parameters of the Hill functions and linear ARX model of each IEG are shown. (DOC) [file pone.0057037.s013.doc]

**Table S1.**

1.cFOS

Hill function

| Input | EC50 | n |
| --- | --- | --- |
| pERK | 0.192 | 2.0779868as a low-pass filter (dash000000000000000000000000000000000000000000000000000000000000000000000000000000000000000000000000 |
| pCREB | 0.776 | 7.68 |

Linear ARX model

| ARX coefficient | | Lag numbers | | | | | | | |
| --- | --- | --- | --- | --- | --- | --- | --- | --- | --- |
|  |  | 0 | 1 | 2 | 3 | 4 | 5 | 6 | 7 |
| Output(A) | cFOS | 1 | -1.648 | 0.675 | -0.011 | -0.108 | -0.118 | 0.4031 | -0.178 |
| Input(B) | f(pERK) |  | -8E-04 | -0.002 | 0.006 | -0.03 | 0.0371 |  |  |
|  | f(pCREB) |  | 0.0374 | -0.051 | 0.0408 | 0.0016 | -0.01 | -0.006 |  |

2.EGR1

Hill function

| Input | EC50 | n |
| --- | --- | --- |
| pERK | 0.287 | 1.16 |

Linear ARX model

| ARX coefficient | | Lag numbers | | | | | | | | | |
| --- | --- | --- | --- | --- | --- | --- | --- | --- | --- | --- | --- |
|  |  | 0 | 1 | 2 | 3 | 4 | 5 | 6 | 7 | 8 | 9 |
| Output(A) | EGR1 | 1 | -1.889 | 0.7985 | -0.272 | 0.8438 | -0.404 | -0.034 | -0.551 | 0.8154 | -0.3032 |
| Input(B) | f(pERK) |  | 0.001 | 0.0078 | -0.041 | 0.0791 | -0.042 |  |  |  |  |

3. cJUN

Hill function

| Input | EC50 | n |
| --- | --- | --- |
| pERK | 0.256 | 1.379 |
| pJNK | 0.399 | 100 |

Linear ARX model

| ARX coefficient | | Lag numbers | | | | | | | | |
| --- | --- | --- | --- | --- | --- | --- | --- | --- | --- | --- |
|  |  | 0 | 1 | 2 | 3 | 4 | 5 | 6 | 7 | 8 |
| Output(A) | cJUN | 1 | -1.297 | -0.033 | 0.07 | 0.4919 | -0.094 | -0.274 | -0.038 | 0.0033 |
| Input(B) | f(pERK) |  | 0.006 | 0.0118 | -0.013 | 0.0033 | -0.021 | 0.0472 | -0.02 |  |
|  | f(pJNK) |  | -0.013 | 0.0115 | 0.0221 | -0.011 | -0.009 | -0.033 | 0.0329 | 0.0057 |

4. JUNB

Hill function

| Input | EC50 | n |
| --- | --- | --- |
| pCREB | 0.732 | 2.24 |
| cFOS | 0.621 | 100 |

Linear ARX model

| ARX coefficient | | Lag numbers | | | | | | | | |
| --- | --- | --- | --- | --- | --- | --- | --- | --- | --- | --- |
|  |  | 0 | 1 | 2 | 3 | 4 | 5 | 6 | 7 | 8 |
| Output(A) | JUNB | 1 | -1.245 | 0.2094 | -0.475 | 0.3308 | 0.3319 | -0.36 | 0.2821 | -0.069 |
| Input(B) | f(pCREB) |  | 0.0214 | -0.019 |  |  |  |  |  |  |
|  | f(cFOS) |  | 0.0274 | -0.027 | 0.027 | -0.027 |  |  |  |  |

5. FOSB

Hill function

| Input | EC50 | n |
| --- | --- | --- |
| cFOS | 0.495 | 1.993 |

Linear ARX model

| ARX coefficient | | Lag numbers | | | | |
| --- | --- | --- | --- | --- | --- | --- |
|  |  | 0 | 1 | 2 | 3 | 4 |
| Output(A) | FOSB | 1 | -1.487 | 0.4137 | -0.057 | 0.0704 |
| Input(B) | f(cFOS) |  | 0.0074 |  |  |  |

**Table S1.** The parameters of the Hill functions and linear ARX model of each IEG are shown.
